# Contribution of Tibetan Plateau ecosystems to local and remote precipitation through moisture recycling

**DOI:** 10.1111/gcb.16495

**Published:** 2022-11-11

**Authors:** Yan Li, Ru Xu, Kun Yang, Yanxu Liu, Shuai Wang, Sha Zhou, Zhao Yang, Xiaoming Feng, Chunyang He, Zhengjie Xu, Wenwu Zhao

**Affiliations:** ^1^ State Key Laboratory of Earth Surface Processes and Resources Ecology Beijing Normal University Beijing China; ^2^ Institute of Land Surface System and Sustainable Development Faculty of Geographical Science, Beijing Normal University Beijing China; ^3^ Ministry of Education Key Laboratory for Earth System Modeling, Department of Earth System Science Institute for Global Change Studies, Tsinghua University Beijing China; ^4^ Pacific Northwest National Laboratory Richland Washington USA; ^5^ State Key Laboratory of Urban and Regional Ecology, Research Center for Eco‐Environmental Sciences Chinese Academy of Sciences Beijing China; ^6^ University of Chinese Academy of Sciences Beijing China; ^7^ Key Laboratory of Environmental Change and Natural Disasters, Ministry of Education Beijing Normal University Beijing China; ^8^ Academy of Disaster Reduction and Emergency Management Ministry of Emergency Management and Ministry of Education Beijing China

**Keywords:** ecosystem service, evapotranspiration, moisture recycling, precipitation regulation, Tibetan Plateau

## Abstract

The ecosystems of the Tibetan Plateau (TP) provide multiple important ecosystem services that benefit both local populations and those beyond, such as through climate regulation services on precipitation for East Asia and China. However, the precipitation regulation service of the TP ecosystems for supplying moisture and maintaining precipitation is yet to be evaluated. In this study, we used the moisture recycling framework and a moisture tracking model to quantify the precipitation regulation services of TP ecosystems for their contribution to precipitation. We found TP ecosystems contributed substantially to local and downwind precipitation, with a contribution of 221 mm/year for the TP and neighboring areas through evapotranspiration (ET) (104 mm/year through transpiration), declined to <10 mm/year for eastern China and other surrounding countries. Among ecosystem types, grassland contributed most to precipitation, followed by barren and snow lands, forests, and shrublands. In terms of seasonality, precipitation contribution from TP ecosystems was greater in summer months than in non‐summer months for western China, while the opposite was true for eastern China—although the magnitude was much smaller. Over the past two decades, the significant ET increases in TP translated to a widespread increase in precipitation contribution for TP and downwind beneficiary regions from 2000 to 2020. Our study provides a quantitative way to understand the precipitation regulation services of TP ecosystems through moisture recycling, substantiating their key role to maintain precipitation and the water cycle for downwind regions—effectively acting as an ecological safeguard that could be perceived by the public.

## Graphical abstract

We used the moisture recycling framework to quantify the precipitation regulation services of ecosystems of the Tibetan Plateau (TP) for their contribution to precipitation. TP ecosystems contributed substantially to local and downwind precipitation, with a contribution declining from TP and neighboring areas to eastern China and other surrounding countries. TP ecosystems effectively act as an ecological safeguard to maintain precipitation and the water cycle for downwind regions.

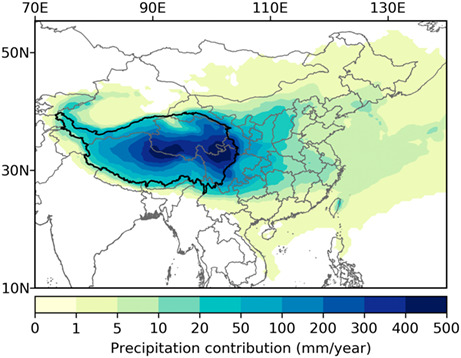

## 1 INTRODUCTION

As the world's highest plateau, the Tibetan Plateau (TP) hosts a variety of ecosystems ranging from shrublands and forests in lowland areas to the unique alpine meadows and steppes at high altitudes (Shen et al., 2022). These diverse ecosystem types provide critical ecosystem services such as biodiversity and soil/water retention (Jiang et al., 2020), delivering a multitude of local benefits. There are other types of services, such as climate regulation, that regulates temperature, precipitation, and other biologically mediated climatic processes at both global and local levels, thus having both local and nonlocal benefits (Costanza, 2008; Costanza et al., 1997). Unlike the plateau's orographic and heating effects on the modern climate (Yang et al., 2020), climate regulation services delivered by ecosystems are closely tied to biogeochemical (e.g., carbon cycle) and biophysical processes. For biogeochemical regulation, plants in TP act as an important carbon sink (Wei et al., 2021), and permafrost also stores a substantial amount of organic carbon (Wang et al., 2020). For biophysical regulation, plant growth in TP exerts a cooling effect by enhanced evaporation (Shen et al., 2015). Recently, significant advances have been made in quantifying the climate regulation service on temperature (Shen et al., 2015; Windisch et al., 2021). Nevertheless, there is limited knowledge regarding the climate regulation service of ecosystems on precipitation resulting from their effects on atmospheric circulation and moisture (Keys et al., 2016).

The moisture recycling process offers a framework to understand and characterize ecosystems' contribution to precipitation (Staal et al., 2018; te Wierik et al., 2021), which is increasingly considered to be an important ecosystem service (Keys et al., 2016). The water evaporated from ecosystems in the source region flows outward through the atmospheric circulation and eventually falls out as precipitation in the downwind sink region (Gimeno et al., 2012; Hoek van Dijke et al., 2022; Tuinenburg et al., 2022). Through this moisture recycling process, the upwind ecosystems thus provide a critical ecosystem service to maintain and sustain precipitation for downwind regions (Mu et al., 2021; O'Connor et al., 2021; Pranindita et al., 2022), that is, the climate regulation service on precipitation through moisture recycling (referred hereafter as precipitation regulation for short)—similar to vegetation‐regulated moisture recycling proposed by Keys et al. (2016). Therefore, places that receive moisture from ecosystems upwind are the beneficiaries of such ecosystem services.

Previous atmospheric and hydrological studies revealed the significant contribution of TP to precipitation over East Asia, identifying its central role in modulating precipitation regimes of its own and downstream areas (Chen et al., 2012; Li et al., 2016; van der Ent et al., 2010). In TP, surface evaporation was estimated to be 65% of total precipitation (Curio et al., 2015). However, only 20%–30% of total precipitation in TP was formed by local evaporation (precipitation recycling ratio [PRR]; Yang et al., 2022), suggesting that more than 50% of evapotranspiration (ET) in TP was transported and formed precipitation outside of TP. These findings provided solid evidence to substantiate the precipitation regulation service of TP through moisture recycling with influences extending much beyond its geographical border. It also revealed that East Asia, one of the most populated regions in the world, is the beneficiary of such services. However, the precipitation regulation service of TP ecosystems has rarely been quantified before. Since the provider and beneficiary of the service are spatially decoupled, it is still unclear how much precipitation in the beneficiary regions originates from TP ecosystems. This knowledge gap also prevents people in the beneficiary regions from comprehending the importance of this service and its faraway provider.

For downwind societies whose precipitation is dependent on this service, maintaining the integrity and functioning of upwind ecosystems is therefore critical to the sustainable delivery of precipitation regulation services (Keys et al., 2018, 2019; Keys & Wang‐Erlandsson, 2018). However, TP ecosystems are highly sensitive to global changes and have experienced significant changes due to climate warming and land‐use changes (Sun et al., 2012). For example, the degradation of alpine meadows and steppes (Wang et al., 2017) threatened the provision of ecosystem services (Hopping et al., 2018). Meanwhile, a number of ecological projects have been implemented in TP since 2008 to safeguard the ecological structure, processes, and patterns of fragile alpine ecosystems against natural and anthropogenic stresses (Gao et al., 2009). These projects covered other ecosystems, such as forest and grassland, and imposed policies including establishing nature reserves, returning pasture to grassland, controlling soil erosion, etc. The first assessment in 2015 showed that these projects reached their designed goals of stabilizing the alpine ecosystem structure and improving their ecosystem services (Wang et al., 2017). By comparing ecosystem changes before and after the project, follow‐up assessment confirmed the positive effects of ecological projects in TP for increasing forest area and vegetation coverage, decreasing desert area, reducing grassland degradation, and improving ecosystem services in water conservation, carbon sinks, and sand fixation, especially in the local regions of the project (Huang et al., 2018, 2019). Spatial comparison analyses also indicated that protected areas in TP effectively reduced deforestation rate (Shen et al., 2021), and promoted vegetation greenness and productivity relative to non‐protected areas (Hua, Zhao, Cherubini, et al., 2022). These ecosystem changes, driven either by natural or human activities (e.g., ecological restoration), would affect ET (Chen et al., 2022; Yu et al., 2020; Zheng et al., 2022), influencing its precipitation regulation capability and thus having regional consequences. However, how much different ecosystems and ecological projects contribute to the precipitation regulation service of TP and how it might be affected by climate/and ecosystem changes remain uncertain.

Quantifying precipitation regulation services became possible with the theory of moisture recycling (van der Ent et al., 2010) and the continued development of analytical and numerical moisture tracking techniques (Chen et al., 2012; Dominguez et al., 2020, 2022; Insua‐Costa & Miguez‐Macho, 2018; Tuinenburg et al., 2020; van der Ent et al., 2010; Wei et al., 2012). These theories and methods allowed for tracing the moisture–precipitation relationship at a large scale, and they have been applied to identify the origin of terrestrial precipitation (Gimeno et al., 2012; van der Ent et al., 2010) [e.g., in dryland (Miralles et al., 2016) and tropical forests (Worden et al., 2021)], characterize processes of moisture recycling (Läderach & Sodemann, 2016; Zemp et al., 2014), inform land cover change impacts (te Wierik et al., 2021; Wang‐Erlandsson et al., 2018; Yang et al., 2019), and guide ecosystem (Creed et al., 2019; Hoek van Dijke et al., 2022; Tuinenburg et al., 2022) and water management (Keys et al., 2017, 2018; Keys & Wang‐Erlandsson, 2018). These research efforts also enabled tracing the spatial and temporal connections of moisture and precipitation in TP (Chen et al., 2012; Gao et al., 2020; Li et al., 2016; Xu & Gao, 2019; Zhang et al., 2017). However, the typical high‐resolution moisture flow simulations (e.g., those conducted by three‐dimensional trajectory‐based analytical models or numerical tracers embedded in climate models) require large data input and high computation resources, which may preclude their wide applications. A recent study created a high‐resolution and global‐scale dataset of the pairwise spatial links of atmospheric moisture–precipitation connections (Tuinenburg et al., 2020), thereby offering a unique opportunity to quantify the precipitation regulation service of TP ecosystems.

In this study, we used the moisture recycling framework and the high‐quality moisture tracking dataset to quantify the precipitation regulation service of TP ecosystems and investigated their spatial–temporal variations. Since ecosystems influence precipitation through multiple pathways, here we only considered precipitation regulation services through moisture recycling, which was quantified as the contribution of TP ecosystems to precipitation through ET and transpiration (T) supply. We also investigated how precipitation regulation services were affected by the ET changes in TP over the past two decades.

## 2 MATERIALS AND METHODS

### 2.1 Methods

#### 2.1.1 Moisture tracking with the UTrack model

The moisture trajectory dataset was produced by simulations of a Lagrangian moisture tracking model “UTrack‐atmospheric‐moisture” (UTrack; Tuinenburg & Staal, 2020) that tracks the moisture flows between each pair of grid cells across the global land. Driven by ERA5 reanalysis data, which have a temporal resolution of 1 h and a spatial resolution of 0.25°, the model was able to perform high‐resolution moisture tracking at the global scale.

Specifically, moisture tracking with UTrack involved three steps. First, at each time step (0.1 h), ET from the land surface was divided into moisture parcels which were then released within the atmospheric column. Second, the released parcels were tracked through time across the three‐dimensional space using wind speed and direction from ERA5 data and a probabilistic profile for vertical mixing; the location and moisture content of parcels were updated at every time step. Third, a fraction of the moisture in a moisture parcel was allocated to rainfall at the location of the parcel based on ERA5 precipitation data, assuming all moisture has the same probability of raining out. Each air parcel was tracked for 30 days or until 99% of its moisture rained out. For more details about the UTrack model and moisture tracking, please refer to Tuinenburg et al. (2020) and Tuinenburg and Staal (2020).

The generated moisture trajectory dataset provides spatial connections between evaporation from a source cell and precipitation in a target cell for every global land grid (Tuinenburg et al., 2020). It consists of monthly climatological mean moisture flow for 2008–2017 expressed as the fractions of evaporation allocated to rainfall (ET to P fraction), with a spatial resolution of 0.5°.

#### 2.1.2 Estimate precipitation contribution based on moisture trajectory data

**FIGURE 1:**
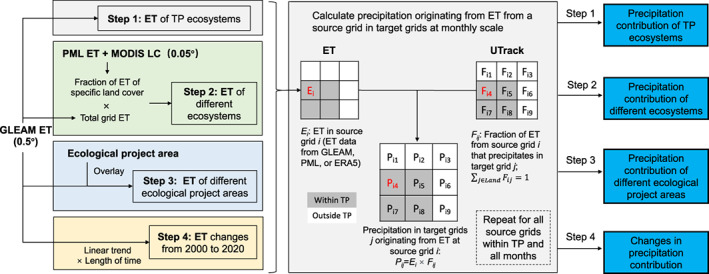
Diagram of the workflow of this study.

The workflow of this study was illustrated in Figure 1. The core process is to combine gridded ET data and the moisture trajectory data to estimate precipitation originating from a source grid cell (shown in grey‐shaded box). For a given source cell *i*, we obtained the grid ET (*E*_*i*_) and the fractions of its ET that form precipitation in target grid cells (*F*_*ij*_, a 2D array). Here, the global sum of *F*_*ij*_ was equal to 1 because water evaporated from the source grid *i* eventually rained out:

(1) $$\sum F_{\mathrm{ij}}=1$$

where *i* is the index of the source grid and *j* ∈ all grids on the earth's surface. Therefore, precipitation (*P*_*ij*_) originating from a source grid cell *i* in target cell *j* can be derived by multiplying *E* of the source cell (*E*_*i*_) by the corresponding ET to P fractions (*F*_*ij*_):

(2) $$P_{\mathrm{ij}}=E_{i}\times F_{\mathrm{ij}}$$

where *i* ∈ all source grids within the TP. The calculation was done for all source grid cells within the TP to obtain the contribution of TP ecosystems to precipitation ($\sum_{i\in \mathrm{TP}}\sum_{j\in \text{earth}}P_{\mathrm{ij}}$). The precipitation contribution was estimated for each month, and the sum of monthly contributions gave seasonal and annual contributions. Since the moisture trajectory produced by UTrack represents a climatological mean state of moisture flow for 2008–2017, in step 1, we used the multi‐year monthly mean ET or T of the same period from GLEAM ET data to estimate the precipitation contribution of TP ecosystems as a whole through ET (*P*_ET_) and transpiration (*P*_T_), respectively. The calculation described above was then applied to steps 2–4, combined with their specific ET to produce precipitation contribution with specific purposes.

In step 2, we separated evaporated water sources by ecosystem types. The UTrack model did not distinguish moisture sources, so water from evaporation/transpiration or different ecosystems was mixed within a grid cell and shared the same trajectory. We used the Penman–Monteith–Leuning (PML) ET dataset (Zhang, 2020) and the Moderate Resolution Imaging Spectroradiometer (MODIS) land‐cover data with a spatial resolution of 0.05° to match the ET of a pixel and its land cover type at 0.05° (ET value was attributed to its land cover type at 0.05°). The monthly shares of different ecosystem types to the grid total ET ($F_{\mathrm{LC}}^{\mathrm{ET}}$) were calculated at a resolution of 0.5° to approximate the ecosystem share of ET within a grid. The fraction of ET of each ecosystem ($F_{\mathrm{LC}}^{\mathrm{ET}}$) derived from PML data at 0.5° multiplied by the grid ET (or T) from GLEAM (ET_grid_) at 0.5° yielded ET (or T) of different ecosystems (ET_LC_):

(3) $${\mathrm{ET}}_{\mathrm{LC}}=F_{\mathrm{LC}}^{\mathrm{ET}}\times {\mathrm{ET}}_{\text{grid}}$$

The resulting ET and T of different ecosystems enabled the estimation of precipitation contribution of different ecosystems in TP.

**FIGURE 2:**
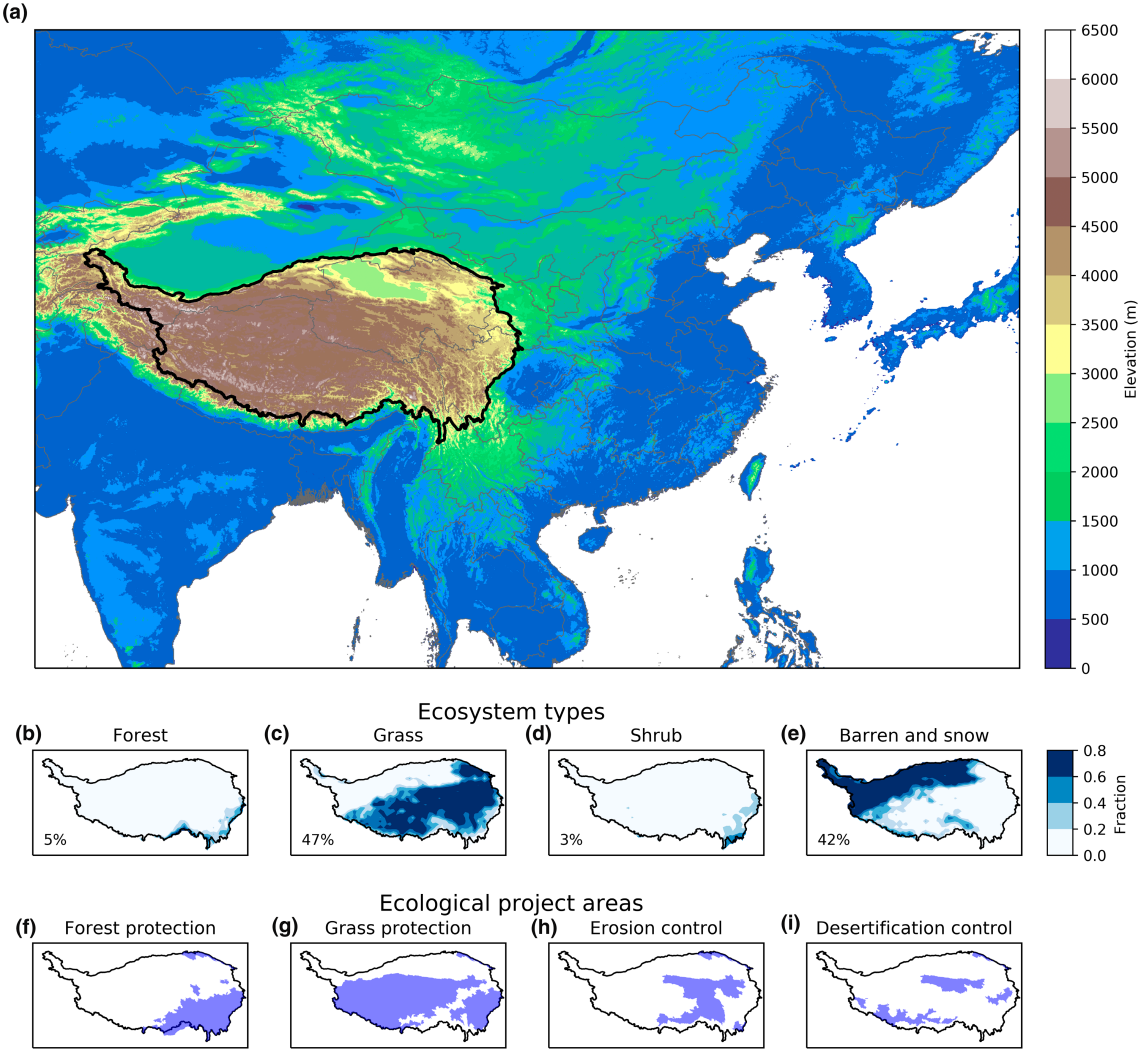
The geographical location of the Tibetan Plateau [TP, black line in (a)], its major ecosystem types (b–e), and ecological project areas (f–i) are shown on the map. Numbers on the second row denote the areal fraction of different ecosystems in TP (other types are not shown due to their small area). Map lines delineate study areas and do not necessarily depict accepted national boundaries.

Ecosystem types were characterized by five groups consisting of combined land cover classes of the International Geosphere‐Biosphere Programme (IGBP) classification of forests (1–5), shrubs (6–9), grass (10), barren and snow (15 and 16), and others (11–14) (Figure 2). The mean ET of an ecosystem type ($\bar{{\mathrm{ET}}_{\mathrm{LC}}}$) can be calculated by the sum of ecosystem ET (ET_LC_) divided by the sum of its areal fraction (*F*_LC_) in TP, both at 0.5°:

(4) $$\bar{{\mathrm{ET}}_{\mathrm{LC}}}=\sum {\mathrm{ET}}_{\mathrm{LC}}/\sum F_{\mathrm{LC}}$$

Since there have been different ecological projects implemented in TP (e.g., forest protection, see details in Section 2.2.3), in step 3, we overlaid the boundary of ecological projects in TP with ET data to extract ET of each project area. The resulting ET was used to estimate the precipitation contribution of different ecological project areas in TP.

In step 4, we estimated linear trends in ET (or T) for each month from 2000 to 2020 to derive their changes over the 21 years in TP (trend × length of time). By applying the climatological moisture trajectories of 2008–2017 to the whole study period of 2000–2020, changes in ET‐contributed precipitation can be obtained by passing ET (or T) changes to the precipitation contribution calculation processes.

### 2.2 Other data

#### 2.2.1 Evapotranspiration and land cover data

**FIGURE 3:**
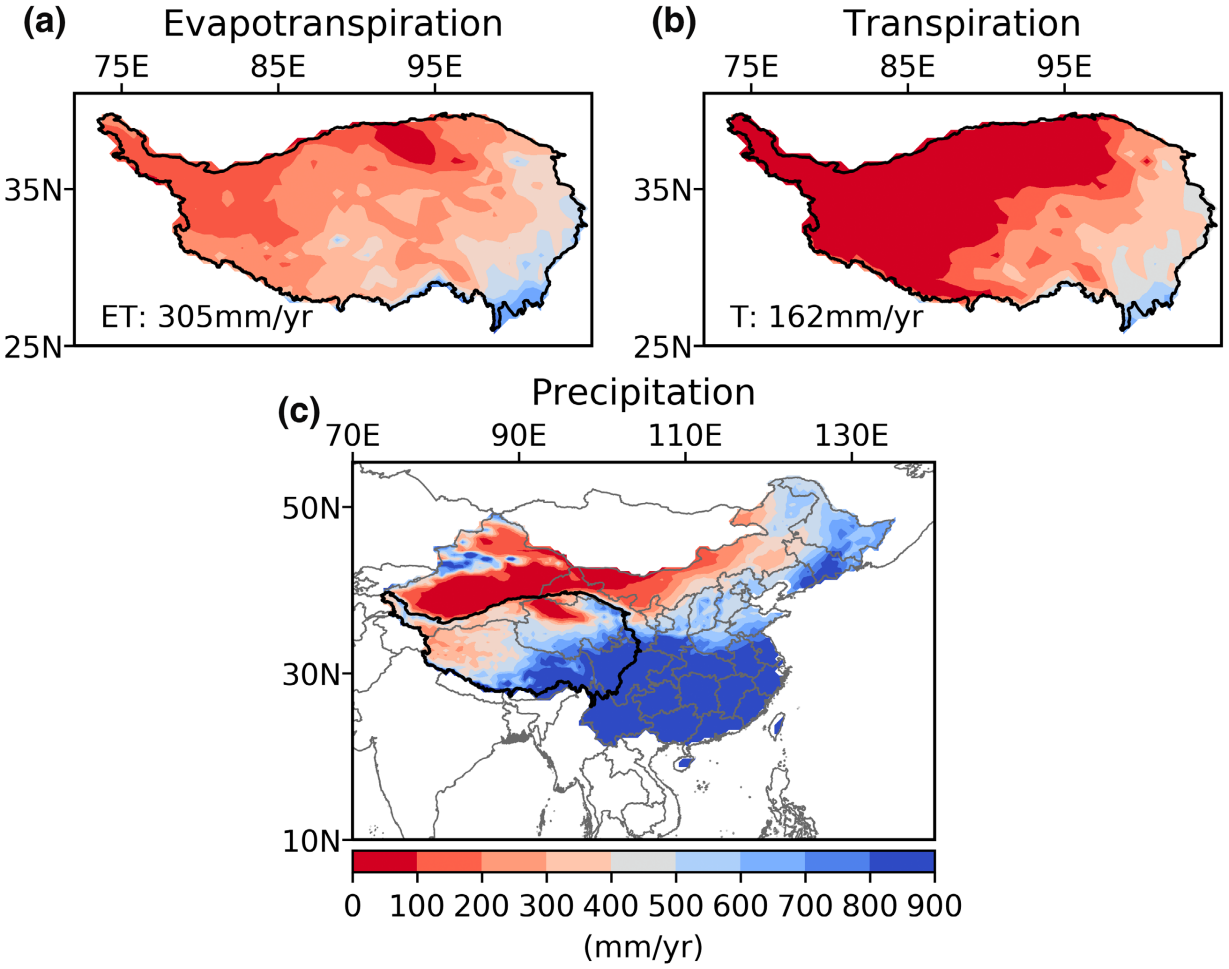
Annual mean evapotranspiration (ET) (a) and transpiration (T) (b) in the Tibetian Plateau from GLEAM data, and annual mean precipitation in China from 2008 to 2017 from ERA5 data (c). Map lines delineate study areas and do not necessarily depict accepted national boundaries.

The GLEAM ET dataset (v3.5a), produced based on satellite and reanalysis data, provides monthly actual ET and transpiration (T) for 1980–2020 at a spatial resolution of 0.5° (Martens et al., 2017; Figure 3a,b). We chose GLEAM ET data because of its good performance in TP (Liu, 2018). The mean actual evaporation and transpiration from 2008 to 2017 were used to represent the amount of water evaporated by ecosystems on the TP. To examine uncertainty associated with ET data, alternative ET datasets, including PML and ERA5 ET from 2008 to 2017, were used to substitute the GLEAM ET to estimate precipitation contribution with the same calculation procedure described in step 1 of Section 2.1.2. The 8‐day PML ET data at 0.05° (Zhang, 2020) were produced by using a coupled diagnostic biophysical model (PML‐V2) that takes MODIS data as inputs with an improved performance than the MODIS ET product (Zhang et al., 2016, 2019). Additionally, the MODIS land cover data in 2008 (MCD12Q1, 0.05°, downloaded from https://earthexplorer.usgs.gov/) were used to estimate the areal fractions of different ecosystems and their ET fractions at 0.5°.

#### 2.2.2 Precipitation data

Precipitation data were derived from ERA5‐Land, including monthly averaged total precipitation at 0.1° resolution from 1950 onward. We selected ERA5 data because it outperformed other gridded precipitation datasets for long‐term spatiotemporal patterns over the whole TP (Yuan et al., 2021). The original 0.1° was bilinearly resampled to 0.5° using the *remapbil* of Climate Data Operators (CDO) to match the resolution of other datasets. The mean precipitation from 2008 to 2017 (shown in Figure 3c) was used to contextualize the precipitation contribution relative to local precipitation.

#### 2.2.3 Ecological project area data in the TP

Four major ecological projects in TP were considered, including forest protection, grassland protection, erosion control, and desertification control (Figure 2). Data sources of ecological projects were compiled from ecological project plans and surveys of departmental‐level administrations in these regions (Wei, 2019). Ecological project plans include the Tibet ecological security barrier protection and construction projects, Sanjiangyuan Nature Reserve ecological protection and construction projects, various ecological protection and construction projects in the Hengduan Mountains, and the Qilian Mountains comprehensive management of ecological protection and construction projects. The map data of the boundary of ecological projects were created by vectorizing different project areas into county‐level distribution maps. Due to the limitation of data sources, the specific location of ecological project within a county was unavailable. Ecological project area refers to areas (county‐level) in which ecological projects have been implemented. Noting that different ecological projects were spatially overlapped because multiple projects could occur in a county. Therefore, precipitation contribution estimated for each ecological project area included all ecosystem types and parts of other project areas within the boundary. Due to their different definitions, precipitation contributions estimated for different ecological project areas and ecosystem types were not comparable.

## 3 RESULTS

### 3.1 Precipitation regulation service of TP ecosystems

**FIGURE 4:**
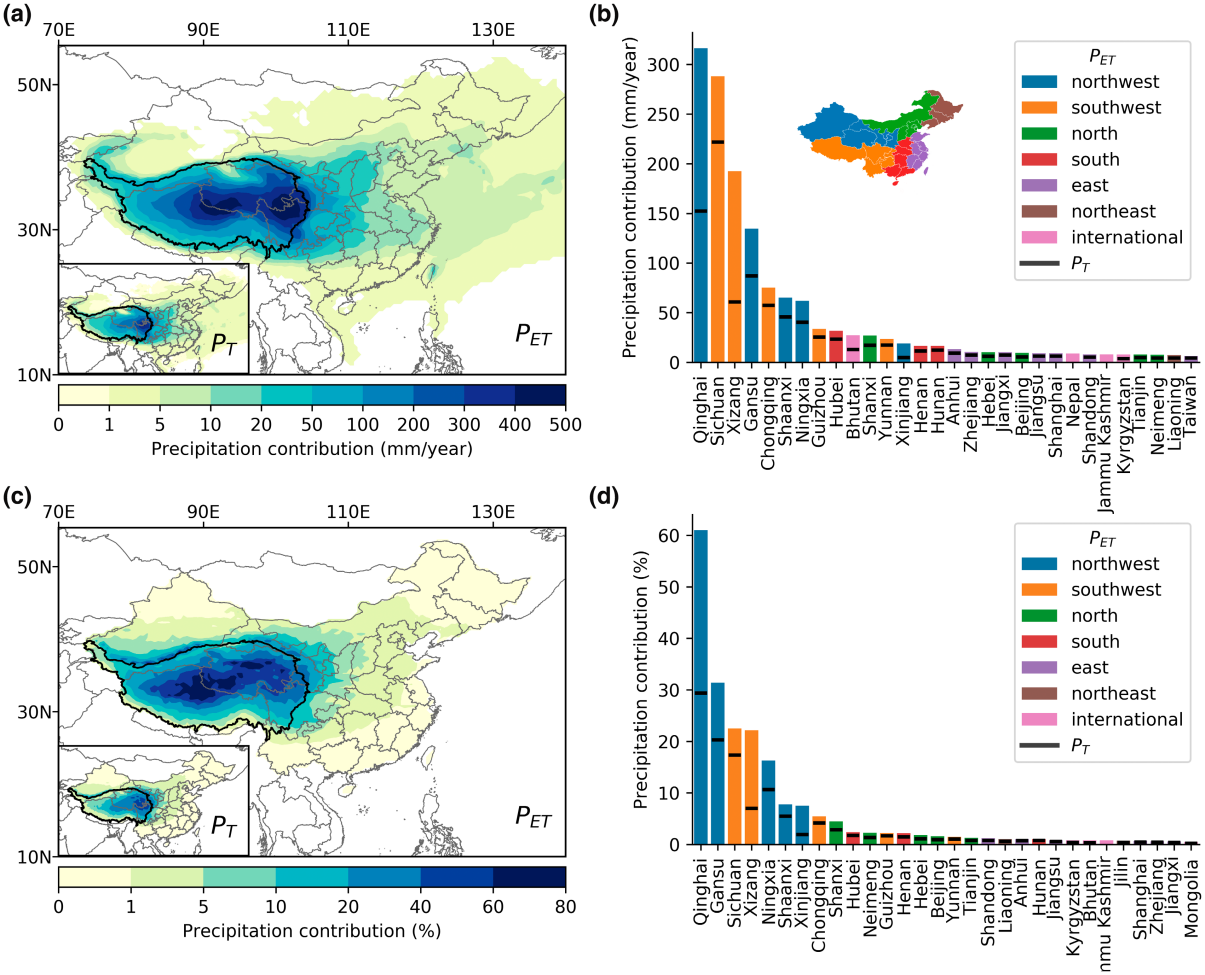
Contribution of Tibetan Plateau ecosystems to annual precipitation through evapotranspiration (*P*_ET_) and transpiration (*P*_T_). The spatial pattern and regional statistics of absolute (a, b) and relative precipitation contribution (c, d). Note that areas with absolute precipitation contribution <1 mm/year were masked on (a). The relative contribution was calculated as precipitation contribution divided by local precipitation of ERA5. Only the top 30 regions are shown in the bar chart. Provincial‐level administrative units in China are grouped into six sub‐regions indicated on the map of (b). Map lines delineate study areas and do not necessarily depict accepted national boundaries.

Figure 4 shows the precipitation regulation service of TP ecosystems, expressed as their contribution to precipitation from 2008 to 2017. Results show that TP ecosystems contributed substantially to precipitation both within and outside TP, with a larger contribution through ET (*P*_ET_) than T (*P*_T_). Spatially, there was an apparent gradient from high contribution over the central‐eastern TP (*P*_ET_ > 500 mm/year) to moderate in neighboring areas (*P*_ET_: 50–100 mm/year), low in central China (*P*_ET_ < 50 mm/year), and eventually negligible in eastern China (*P*_ET_ < 5 mm/year) (Figure 4a). This decreasing pattern from local to distant regions was mainly caused by the prevailing westerly winds over China, which was more evident when breaking precipitation contribution into administrative units (Figure 4b; Table S1). Specifically, Qinghai (316 mm/year), Sichuan (288 mm/year), and Xizang (192 mm/year) in China, whose lands constitute the TP, received the largest precipitation contribution from TP ecosystems through ET (*P*_ET_). The regional ranking was different for *P*_T_, where Sichuan (222 mm/year) was the leading province. For regions far away from TP, such as northeastern China, the precipitation contribution was rather limited (*P*_ET_ < 1.5 mm/year). Notably, neighboring countries/regions adjacent to TP, such as Bhutan (27 mm/year), Nepal (9 mm/year), Kashmir (8 mm/year), and Kyrgyzstan (8 mm/year), also received precipitation benefits from TP ecosystems through ET.

To contextualize precipitation contribution, we calculated the relative contribution by dividing it by local precipitation from ERA5 (Figure 4c). The largest relative contribution of *P*_ET_ appeared in central TP, accounting for about >70% of local precipitation. The precipitation contribution through ET averaged over the whole TP was 221 mm/year, suggesting a relative contribution and an equivalent PRR of 29%. In contrast, the precipitation contribution through T was smaller over the TP (104 mm/year for *P*_T_) and the relative contribution was 14%. In terms of regional ranks, the relative contribution differed from the absolute contribution, where Qinghai (61%), Gansu (31%), Sichuan (23%), and Xizang (22%) were the leading regions of China for *P*_ET_ (Figure 4d). The relative contribution through ET decreased to 5%–20% in adjacent areas and further down to <5% in eastern China. The eastward decreasing relative contribution revealed a critical role of precipitation regulation service for TP and adjacent inland areas but a minor role for distant regions whose primary moisture sources come from the ocean and monsoon (not shown).

### 3.2 Precipitation regulation services of different ecosystems and ecological project areas in the TP

To investigate the precipitation regulation service of different ecosystems in TP, we estimated their precipitation contributions separately (Table 1). Although different ecosystems had different ET, which determines the amount of moisture ecosystems released to the atmosphere per unit area, the magnitude of precipitation contribution of different ecosystems primarily reflected their spatial extent because a larger area corresponds to a greater total amount of ET. Specifically, the mean ET decreased from forests (973 mm/year), grassland (400 mm/year), barren and snow lands (132 mm/year) to shrubland (88 mm/year). However, grassland, occupying 47% area of TP, was the ecosystem that contributed most to TP precipitation through ET (138 mm/year). Barren and snow lands in northwest TP (42% area) with little vegetation cover made a considerable precipitation contribution of 65 mm/year in TP. Forests covering the east edges of TP (5% area) contributed 9.1 mm/year precipitation in TP. Shrub (3% area) overlapped with forests and had only about 5.2 mm/year contribution in TP.

**TABLE 1:** Contributions of different ecosystems and ecological project areas in TP to precipitation through ET in different regions of China (unit: mm/year)

Region	Precipitation contribution by ecosystem types (mm/year)					Precipitation contribution by ecological project areas (mm/year)			
	Forest	Shrub	Grass	Bare/snow	Other	Grassland protection	Forest protection	Erosion control	Desertification control
Qinghai	3.8 (0.73%)	2.6 (0.5%)	223 (43%)	80 (15%)	3.4 (0.65%)	241 (46%)	93 (18%)	149 (29%)	112 (22%)
Sichuan	46 (3.6%)	33 (2.6%)	192 (15%)	15.0 (1.2%)	1.7 (0.13%)	222 (17%)	253 (20%)	135 (11%)	45 (3.5%)
Xizang	4.8 (0.55%)	0.93 (0.11%)	106 (12%)	77 (8.9%)	0.18 (0.02%)	161 (19%)	42 (4.8%)	41 (4.8%)	65 (7.5%)
Gansu	8.7 (2.0%)	6.1 (1.4%)	91 (21%)	23.0 (5.3%)	5.6 (1.3%)	87 (20%)	71 (17.0%)	51 (12%)	38 (8.8%)
Chongqing	20 (1.5%)	16 (1.2%)	34 (2.5%)	3.1 (0.23%)	0.97 (0.07%)	53 (3.8%)	67 (4.9%)	30 (2.1%)	7.9 (0.57%)
Shaanxi	9.3 (1.1%)	6.2 (0.75%)	40 (4.8%)	6.5 (0.78%)	2.8 (0.34%)	47 (5.6%)	43 (5.2%)	23 (2.7%)	16 (1.9%)
Ningxia	4.3 (1.1%)	3.0 (0.79%)	41 (11%)	9.3 (2.4%)	4.2 (1.1%)	38 (10.0%)	31 (8.3%)	23 (6.0%)	17 (4.6%)
Guizhou	11 (0.71%)	8.9 (0.58%)	12 (0.81%)	1.2 (0.08%)	0.28 (0.02%)	18 (1.2%)	31 (2.0%)	11 (0.72%)	2.5 (0.17%)
Hubei	7.2 (0.54%)	5.5 (0.41%)	16.0 (1.2%)	2.3 (0.17%)	0.71 (0.05%)	22 (1.6%)	25 (1.9%)	12 (0.89%)	4.8 (0.36%)
Shanxi	2.4 (0.39%)	1.6 (0.27%)	17 (2.8%)	4.6 (0.76%)	1.5 (0.25%)	17 (2.8%)	15 (2.4%)	10 (1.7%)	7.2 (1.2%)
Yunnan	8.7 (0.54%)	6.3 (0.39%)	7.6 (0.47%)	0.92 (0.06%)	0.09 (0.01%)	8.3 (0.52%)	23 (1.4%)	5.9 (0.37%)	0.9 (0.06%)
Xinjiang	0.0 (0.0%)	0.01 (0.0%)	3.5 (1.4%)	15 (5.9%)	0.48 (0.19%)	5.5 (2.2%)	0.21 (0.08%)	1.2 (0.47%)	1.3 (0.53%)
Henan	2.5 (0.33%)	1.8 (0.24%)	9.6 (1.3%)	2.1 (0.27%)	0.6 (0.08%)	11 (1.5%)	11 (1.4%)	6.1 (0.8%)	3.6 (0.48%)
Hunan	4.3 (0.27%)	3.5 (0.21%)	7.4 (0.46%)	1.0 (0.06%)	0.28 (0.02%)	10 (0.63%)	14 (0.86%)	5.9 (0.36%)	2.1 (0.13%)
Anhui	2.6 (0.2%)	2.0 (0.15%)	7.0 (0.54%)	1.4 (0.11%)	0.33 (0.03%)	8.7 (0.67%)	9.6 (0.74%)	4.9 (0.38%)	2.3 (0.18%)
TP	9.1 (1.2%)	5.2 (0.68%)	138 (18%)	65 (8.4%)	1.7 (0.22%)	169 (22%)	82 (11%)	79 (10%)	66 (8.6%)

*Note*: Numbers in parentheses denote relative contribution (%). Regions are ranked by annual precipitation contribution and only the top 15 regions are shown in the table (the full table is provided in Table S1). A similar table but for precipitation contribution through T (*P*_T_) is shown in Table S2. Note that different ecological project areas have overlap, so their summed precipitation contribution is larger than that of TP. Precipitation contributions estimated for different ecosystem types and ecological project areas are not comparable due to their different definitions.

Abbreviations: ET, evapotranspiration; TP, Tibetian Plateau.

Precipitation contributions of different ecosystems to downstream regions outside of TP also varied, depending on the extent and location of the ecosystem and its distance to TP along the prevailing westerly winds. Grassland was the dominant precipitation contributor for most regions (Table 1). Nevertheless, forests contributed more precipitation through ET than grass in certain regions such as Yunnan due to their proximity to TP along the downwind westerly. As the second largest contributor, barren and snow lands made a prominent contribution in Qinghai (80 mm/year) and Xizang (77 mm/year) through ET.

Although ecological projects in TP were designed for various purposes, they acted to improve the overall ecosystem conditions (e.g., through reversing degradation caused by overgrazing; Huang et al., 2019), promoting vegetation recovery/growth and thereby intensifying ET (Xiao et al., 2021; Zheng et al., 2022). Here, we estimate precipitation contribution from areas implemented with ecological projects to examine their role in precipitation regulation service. The grassland protection area was located in most central‐southern TP, and it contributed to 169 mm/year of *P*_ET_ for TP. This was followed by 82 mm/year from the forest protection area in southeast TP, 79 mm/year from the erosion control area in central‐southern TP, and 66 mm/year from the desertification control area spreading scattered in TP.

Similarly, precipitation contributions from ecological project areas had regionally varying importance. Overall, the grassland protection area was the leading precipitation contributor. The forest protection area made the largest *P*_ET_ in Sichuan (253 mm/year), Chongqing, Guizhou, and Yunnan. Considerable *P*_ET_ were made by the erosion control area in Qinghai (149 mm/year) and Sichuan (135 mm/year) and by the desertification control area in Qinghai (112 mm/year) and Xizang (65 mm/year).

### 3.3 Seasonal variations in precipitation regulation service of TP ecosystems

**FIGURE 5:**
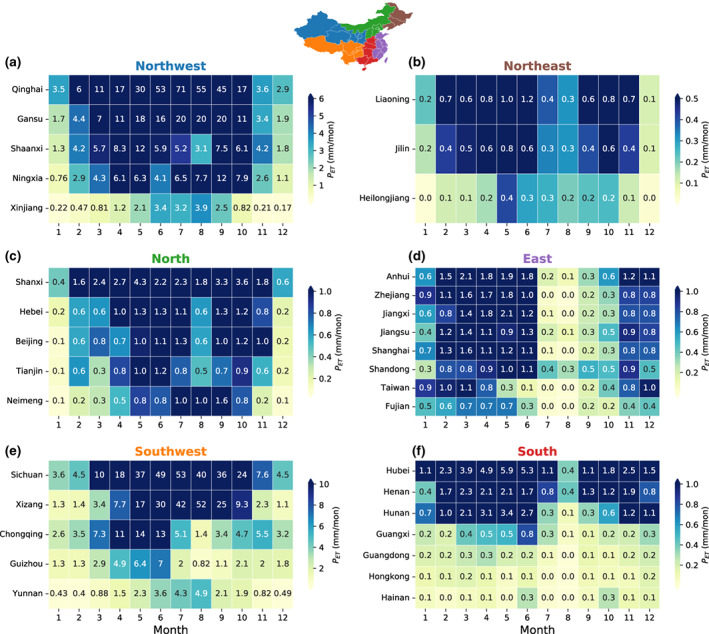
Seasonal variations in the precipitation contribution of Tibetian Plateau ecosystems through evapotranspiration (*P*_ET_) in China. The precipitation contribution through T (*P*_T_) is shown in Figure S1. Provincial‐level administrative units of China are grouped into six sub‐regions (a–f) as shown on the top map, with corresponding color matches the panel title. Numbers annotated on each cell refer to precipitation contribution. Map lines delineate study areas and do not necessarily depict accepted national boundaries.

Seasonal variations in both moisture flow and ET resulted in distinct seasonal variations in the precipitation contribution of TP ecosystems, which was important for seasonally‐sensitive systems (e.g., rainfed agriculture, reservoirs, and livestock; Keys et al., 2016; Figure 5). Generally, for most regions in the western half of China (the left column of Figure 5, including Northwest, North, and Southwest China), precipitation originating from TP ecosystems was greater in summer months than in non‐summer months. For northwest China, which is mainly inland areas with an arid and semi‐arid climate, the summer precipitation contribution was particularly important. *P*_ET_ accounted for 27% of precipitation in Gansu, 11% in Ningxia, and 9.1% in Xinjiang (Table S3, see Table S4 for *P*_T_), and these provinces were ecologically fragile and major production areas for cotton and livestock in China. Such seasonality was more notable in regions receiving more precipitation from TP ecosystems (e.g., Qinghai, Sichuan, and Xizang). For example, precipitation contribution through ET to Qinghai could be as high as 53–71 mm/month in summer and decreased to 2.9–6 mm/month in winter.

For the eastern half of China (the right column of Figure 5, including Northeast, East, and South China), precipitation originating from TP ecosystems showed the opposite seasonality, with a larger contribution in non‐summer months than in summer months. This is likely due to the seasonal changes of prevailing winds of the East Asian monsoon in eastern China: the southeast winds in summer prevent moisture transport from TP to eastern China (Guo & Wang, 2014), while northeast winds in winter favor such transport. However, the precipitation contribution in the eastern half of China was small relative to total precipitation (*P*_ET_ < 5%, Figure 4d; Table S1), indicating a much lower reliance on precipitation supplied by TP ecosystems. This is because moisture evaporated from TP falls out as precipitation along its transportation pathways to eastern China with fewer left while moisture from the ocean is increasing (not shown).

### 3.4 Changes in precipitation regulation service over the last two decades

**FIGURE 6:**
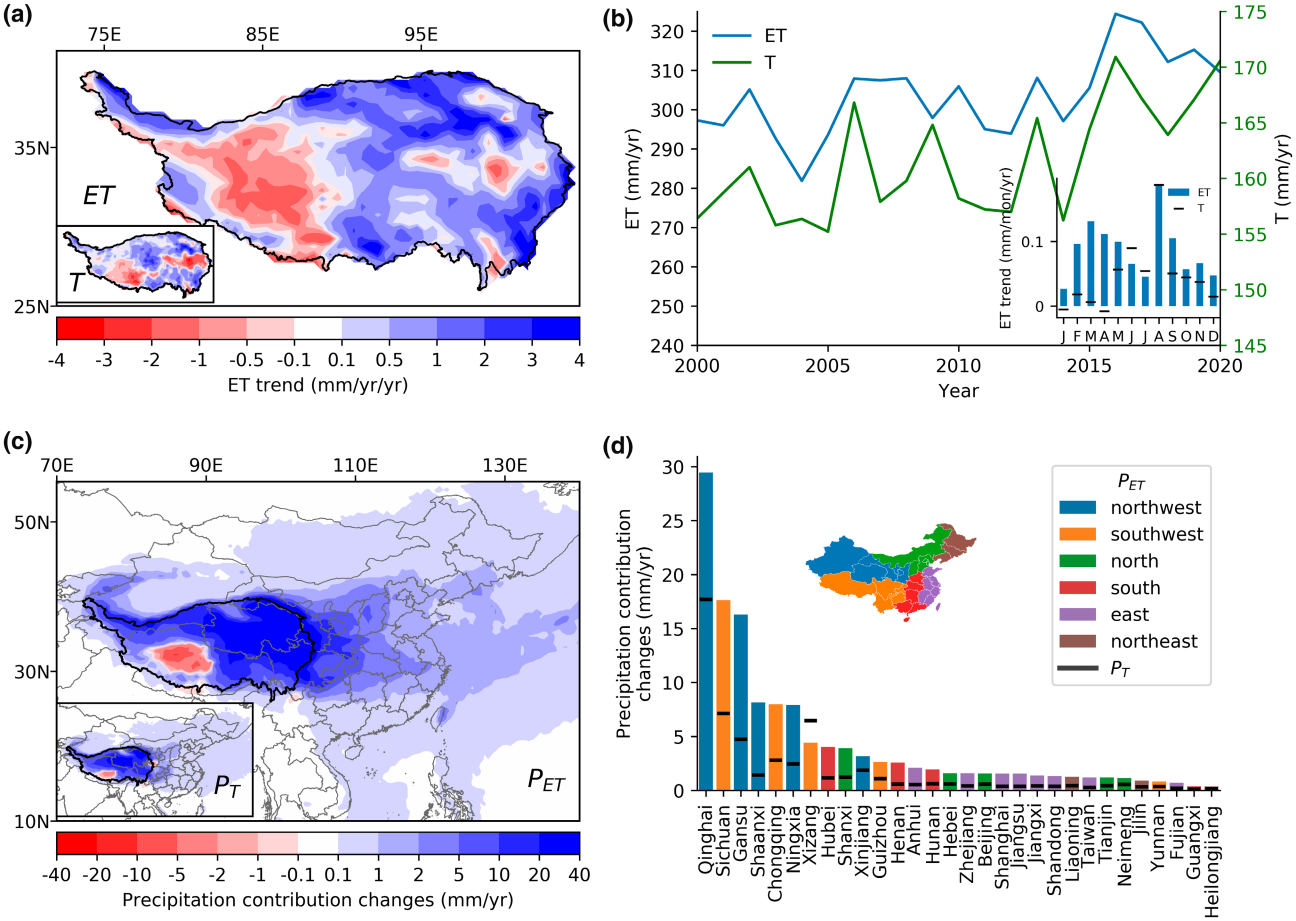
Changes in evapotranspiration (ET) and transpiration (T) and their effects on precipitation contribution of Tibetian Plateau (TP) ecosystems during 2000–2020. (a) Spatial pattern of ET and T trends during 2000–2020 over TP based on GLEAM data. (b) Regional ET and T changes in TP and their monthly trends. (c, d) Changes in ET‐ (*P*_ET_) and T‐contributed precipitation (*P*_T_) and their regional statistics. Only the top 30 regions are shown in the bar chart. Provincial‐level administrative units in China are grouped into six sub‐regions indicated by the inset map of (d). Map lines delineate study areas and do not necessarily depict accepted national boundaries.

Over the past two decades, ET has changed dramatically over the TP. Although there were inconsistencies regarding ET changes over TP (Han et al., 2021; Ma & Zhang, 2022), GLEAM data indicated an overall increasing trend in ET from 2000 to 2020 for most TP, ranging from 0.25 to 6 mm/year, except for a decrease in parts of southwestern TP owing to reduced net radiation (Han et al., 2021; Li et al., 2022; Figure 6a). The increasing ET trends were explained primarily by rising temperature, increasing precipitation, and vegetation greening (Liu et al., 2021; Song et al., 2022). The annual ET trends over TP were estimated to be 1.04 mm/year/year from 2000 to 2020, which translated to an ET increase of 21.9 mm. The ET trends also differed on a monthly scale, with stronger increases in August (~0.2 mm/month/year) and spring months (>0.1 mm/month/year; Figure 6b). By contrast, the annual T trends over TP were 0.55 mm/year/year, equivalent to a T increase of 11.5 mm from 2000 to 2020, which was primarily caused by increasing precipitation and vegetation greening (Ma & Zhang, 2022; Wang et al., 2018).

Evapotranspiration changes, especially the increasing ET in the source regions, were expected to cause changes in the precipitation regulation service of TP ecosystems. Applying the moisture flow climatology between 2008 and 2017 to represent the past ~20 years, we could estimate changing precipitation contribution induced by ET changes through moisture supply (see Section 2.1). As shown in Figure 6c, the increasing ET trends in TP translated to a widespread increase in precipitation contribution in local and remote regions, with a similar decreasing magnitude outward from TP. The ET decrease in southwestern TP (i.e., the grassland in western Xizang) translated to a decreased contribution to local precipitation. At the regional level (Figure 6d), large increases in precipitation contribution were found in Qinghai (29 mm/year for *P*_ET_ and 18 mm/year for *P*_T_), Sichuan (18 mm/year for *P*_ET_ and 7 mm/year for *P*_T_), and Gansu (16 mm/year for *P*_ET_ and 5 mm/year for *P*_T_), while for the rest of China, the increases were below 10 mm/year during the study period.

## 4 DISCUSSION

By using moisture tracking and ET data, we quantified the precipitation regulation service of TP ecosystems and demonstrated their vital contribution to both local and remote precipitation. The contribution to precipitation within the TP is through internal moisture recycling, while the contribution to remote precipitation outside of TP is through atmosphere transportation of evaporated moisture that eventually falls downwind.

The precipitation contribution within the TP through ET (about 221 mm/year) implied a PRR of 29%, which lies in the upper bound of the reported range of 20%–30% (Yang et al., 2022). However, the PRR value should be interpreted with caution because several uncertainty factors were involved in estimating precipitation contribution, including (1) ET and precipitation data and (2) specification of Utrack for moisture flow tracing. First, we found that the precipitation contribution was sensitive to the choices of ET and precipitation datasets (Table 2), depending on both the ET amount and its spatial distribution. The *P*_ET_ and PRR in TP varied substantially from 221 to 298 mm/year and 29% to 39%, respectively, among different ET datasets. As expected, the larger ET in PML and ERA5 data led to higher estimates of *P*_ET_ and PRR than that of GLEAM data. However, ERA5 data made a higher *P*_ET_ than PML (298 vs. 292 mm/year), although their mean ET over the TP was almost the same. This is because higher ET in ERA5 mainly appeared over the western TP. In comparison, higher ET in PML was distributed toward eastern TP, whose moisture is more likely to form precipitation outside of TP due to the westerly winds (Figure S2). As for precipitation, a high‐resolution climate model simulation in TP suggested regional mean precipitation of 702 mm/year (Jiang et al., 2021; Y. Jiang, personal communication, 2022), which was lower than 766 mm/year from ERA5. Adopting the former precipitation would result in even higher PRR in TP (31%–42%). Although ET and precipitation data are critical for such quantification, estimating them accurately is still challenging over TP due to its high elevation with complex topography and insufficient observations. Second, uncertainties in the moisture trajectory were introduced by the inherent simplification and assumption of tracking models and the wind and humidity driving data from ERA5. Additionally, differences in the study period, domain, and a longer tracing length (30 days compared to <15 days in other studies; Chen et al., 2012; Jiang et al., 2017; Xu & Gao, 2019) also contributed to the different reported precipitation contributions in the literature. Though the magnitude of precipitation contribution is subject to significant uncertainty, its spatial pattern, including the spatial extent of regions receiving precipitation from TP and the eastward decreasing contribution, are robust, as the latter is driven by atmosphere circulation and prevailing winds. These results provide a quantitative way to understand the precipitation regulation services of TP ecosystems.

**TABLE 2:** Influence of ET and precipitation datasets on the estimated precipitation contributions over the TP (unit: mm/year)

ET dataset	GLEAM ET	PML ET	ERA5 ET
ET in TP	306	391	391
Precipitation in TP from ERA5	766		
Precipitation contribution in TP	221	292	298
Precipitation recycling ratio (PRR)^a^	28.90%	38.12%	38.90%

Abbreviations: ET, evapotranspiration; TP, Tibetian Plateau.

^a^ PRR is the ratio of precipitation contributed by ET of TP to total precipitation of TP.

The precipitation contribution was estimated based on climatological mean moisture trajectories and ET, ignoring their potential interannual variabilities. Previous studies suggested a stable spatial relationship between the upwind source and downwind sink regions of precipitation across years (Keys et al., 2014; Miralles et al., 2016), reinforcing the robustness of the spatial pattern of precipitation contribution. However, interannual variabilities in moisture trajectory are expected, for example, between EI Nino and La Nina years (Zhao & Zhou, 2021). How precipitation contribution varies across years needs further study as currently, we do not have the yearly moisture tracking data. Moreover, ET has considerable interannual variability, which affects downwind precipitation that relies on upwind moisture supply. It also highlights the importance of ET from the source region to sustain precipitation downwind during dry periods because the upwind changes in ET could dampen or exacerbate drought conditions downwind (Mu et al., 2021; Pranindita et al., 2022; Staal et al., 2018; Wei et al., 2016).

In this study, we only considered the effect of ecosystem ET or T as a moisture source on precipitation, while ecosystems could affect precipitation through other pathways, including the atmospheric circulation processes for moisture transport and precipitation triggering in the downwind sink region (e.g., the convective stability; Wei & Dirmeyer, 2019). Specifically, ecosystems with different biophysical properties directly influence ET in the moisture origin region and the land–atmosphere interactions through surface energy partitioning between sensible and latent heat, which may affect atmospheric circulation processes. We did not differentiate sources of moisture (water from evaporation or transpiration) when tracking moisture. In the UTrack model, evaporated and transpired water is assumed to be well‐mixed, and their differences in the isotopic composition are neglected (e.g., transpired water is heavier than evaporated water; Gimeno et al., 2012). To highlight the role of vegetation, we also estimated precipitation contribution through transpiration (*P*_T_). According to GLEAM data, transpiration accounted for 57% of ET in TP, and the resulting *P*_T_ (104 mm/year) was 47% of *P*_ET_ (221 mm/year) in TP. This indicated that precipitation contribution through T did not follow a simple scaling of T to ET within a given region because the transported moisture is also affected by the spatial distribution of T (e.g., the location of the moisture source on its trajectory). The high T over eastern TP implied more precipitation would form in the downwind region outside TP for *P*_T_. However, the partitioning of ET in TP was uncertain as different datasets provided a wide range of estimates (Lian et al., 2018; Wei et al., 2017). A recent study using the PML model showed that 31% of ET in TP came from plant transpiration, plus 5% from canopy evaporation (Ma & Zhang, 2022). Although the direct contribution of vegetation to ET through T seems to be low, its indirect contribution through vegetation feedback would still be more substantial because the presence of vegetation increases precipitation and ET—particularly compared with bare ground through changing atmospheric circulation (Snyder et al., 2004). The indirect effect is particularly relevant for remote precipitation, which depends on circulation. Moreover, further complications come from moisture transportation being a cascade process involving multiple ET‐precipitation cycles (Staal et al., 2018; Zemp et al., 2014). ET in TP that benefits downwind regions also received precipitation formed by moisture transported from upwind vegetation. Given that the water cycle is shaped by the entangled local and nonlocal interactions between vegetation and the environment (Van Der Ent et al., 2014; Wang‐Erlandsson et al., 2014, 2018), a strict separation of vegetation‐only‐induced ET and its precipitation contribution would be difficult. Quantifying precipitation contribution from ET at the ecosystem level, which includes both biotic and abiotic factors instead of transpiration, would reduce uncertainty in ET partitioning and align with other moisture recycling studies.

Additionally, the ecological projects in TP aimed at improving ecological conditions, such as those implemented over forests and grassland (Hua, Zhao, & Pereira, 2022), could also enhance the precipitation contribution through both direct (increased ET) and indirect mechanisms (land‐atmosphere interaction). There has been ample evidence suggesting that ecological projects improved vegetation and intensified ET (Chen et al., 2022; Yu et al., 2020; Zheng et al., 2022). Some even argued that ecological restoration may risk water resources in TP through increased ET (Xiao et al., 2021). Our results showed that ET originating from TP partially returned to TP, and the rest formed precipitation elsewhere. However, due to lacking data of the specific location of ecological project, we could not assess how much ET changes are attributable to implementing these projects, which requires independent investigation. Further work could improve the evaluation and attribution of ecological projects by collecting data between the implemented and control sites before and after the implementation. Nevertheless, our results demonstrated the magnitude of precipitation contribution from areas implemented with ecological projects and their broad impact on downwind regions beyond their boundary.

Moisture recycling framework suggests that ET changes in the source region like TP could change precipitation contribution to downwind sink regions. This is particularly important as the increase in ET intensified precipitation recycling over the TP from 1979 to 2008 (Guo & Wang, 2014). The observed ET increases shown in Figure 6 were dominated by climate factors instead of ecosystem changes (Ma & Zhang, 2022). The gross ecosystem changes in TP between 2001 to 2020 were less than 2% in terms of areal coverage based on MODIS land cover data (Table S5), suggesting limited impacts on ET changes (Li et al., 2022). Regardless of drivers, translating ET changes to precipitation can inform the possible consequences of the ecosystem‐driven changes (e.g., induced by ecological restoration) in precipitation regulation services. A complete picture of the climate impacts of ecological projects would need to consider full pathways of vegetation‐precipitation interactions by using hydrological, land surface, or regional climate models.

**FIGURE 7:**
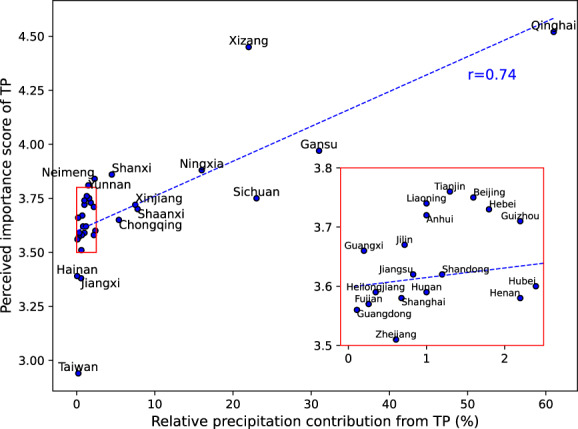
Relationship between relative precipitation contribution from Tibetian Plateau (TP) ecosystems through evapotranspiration (ET) and perceived importance of TP (score from 1 to 5) by more than 20,000 respondents in an online survey (Liu, 2020). Inset shows the zoomed view of the scatters.

The considerable precipitation contributed by TP ecosystems and ecological project areas proves it as a vital ecosystem service for local and remote regions. However, to what extent this invisible service and its benefits, which were rarely quantified before, could be perceived by local residents is still unknown. To investigate this issue, we utilized data from an online survey, “Willingness to Pay for Ecological Resources Protection in Tibetan Plateau” (Liu, 2020). The survey was conducted from March to June 2018 and consisted of 25,696 internet questionnaires covering all provinces in China. Using respondents' answers to the question “how much do you think TP ecosystems are important to the residents of your place of residence” (score from 1 to 5) as a proxy to represent the perceived importance of TP in the Chinese public, we found a strong positive correlation between the relative precipitation contribution through ET (*P*_ET_) and the perceived importance score of TP across different provinces (*r* = .74, Figure 7). This suggested that people in regions that received greater precipitation benefits also perceived a higher importance of the TP. Although these survey participants were unaware of the underlying physical processes (e.g., moisture recycling), their perceived importance coincided with decreasing precipitation contribution in downwind regions (due to westerly winds)—as shown in our results. The consistency between delivered ecosystem service and perception of the beneficiary indicated a causal linkage between the service provider and consumer, although these two were spatially separated. Alternatively, the linkage could be explained by the physical distance to TP (distance measured between centroids), as residents far away from TP would be less concerned with TP ecosystems (*r* = −.72). Regardless, the importance score showed a marginally larger correlation with the relative precipitation contribution than the physical distance to TP. Despite the inherent limitation of survey data (e.g., demographic and sampling biases), these results still help bridge the gap between public perception (which might be driven by intuition) and physical processes related to ecosystem service. The combination of the objective ecosystem service quantity and subjective perception sheds light on the relationship between ecosystem services and human wellbeing (Fu, 2020). The quantified precipitation contribution in our assessment provides physical evidence to support ecological protection in TP, as the avoided ecosystem degradation and improvement sustains delivering services to local and nonlocal regions (Creed et al., 2019). With this knowledge, the changing service caused by ecosystem and climate changes (Figure 6) could be better understood by the public, potentially shifting their perception from intuition‐ to science‐driven understandings—therefore reinforcing their environmental awareness and potentially influence the decision‐making processes within ecosystem management and protection practices (e.g., conservation, restoration, or ecosystem service payment).

## 5 CONCLUSION

Based on the moisture recycling framework, our study quantified the precipitation regulation service of TP ecosystems for supplying moisture and maintaining local and downwind precipitation. Our results revealed a substantial precipitation contribution of the TP to itself (i.e., moisture recycling), outside adjacent provinces of China, and even other countries/regions, with a declined contribution from west to east. Therefore, ecosystems on TP effectively act as an ecological safeguard of precipitation and water cycle for large parts of China and other countries/regions, thus benefiting both local populations and those beyond. The consistency between precipitation benefits and the public's perceived importance of TP ecosystems provided causal evidence linking ecosystem service to human being. Driven by climate changes (melting of snow, ice, and frozen soil) or human activities (land‐use change, grazing, restoration), ecosystems in TP have been experiencing rapid changes in structure, composition, and extent in the past decades and are expected to continue in the future (Zhao et al., 2021). The accompanied ET changes would alter the precipitation regulation service of TP ecosystems for local and remote regions. How precipitation regulation services respond to warming and ongoing ecosystem management and restoration is worth further attention (Hoek van Dijke et al., 2022; Tuinenburg et al., 2022). Despite the uncertainties in data and methods, our attempt to quantify precipitation regulation services advances the understanding of ecosystem services of TP and their contributions to relevant populations. This knowledge is therefore essential for the policy‐making process of ecosystem management and ecological restoration practices in TP.

## AUTHOR CONTRIBUTIONS

Yan Li conceived the study and performed data analysis with inputs from Ru Xu, Kun Yang, and Zhengjie Xu; Yan Li wrote the manuscript with contributions from Kun Yang, Yanxu Liu, Shuai Wang, Sha Zhou, Zhao Yang, Xiaoming Feng, Chunyang He, and Wenwu Zhao.

## 6 CONFLICT OF INTEREST

We declare no conflict of interest of this work.

## Supporting information


Appendix S1

## Data Availability

All code and data needed to reproduce this study are available at Figshare (https://doi.org/10.6084/m9.figshare.20390421).

## References

[gcb16495-bib-0001] Chen, B. , Xu, X.‐D. , Yang, S. , & Zhang, W. (2012). On the origin and destination of atmospheric moisture and air mass over the Tibetan Plateau. Theoretical and Applied Climatology, 110(3), 423–435. 10.1007/s00704-012-0641-y

[gcb16495-bib-0002] Chen, P. , Wang, S. , Song, S. , Wang, Y. , Wang, Y. , Gao, D. , & Li, Z. (2022). Ecological restoration intensifies evapotranspiration in the Kubuqi Desert. Ecological Engineering, 175, 106504. 10.1016/j.ecoleng.2021.106504

[gcb16495-bib-0003] Costanza, R. (2008). Ecosystem services: Multiple classification systems are needed. Biological Conservation, 141(2), 350–352. 10.1016/J.BIOCON.2007.12.020

[gcb16495-bib-0004] Costanza, R. , D'Arge, R. , de Groot, R. , Farber, S. , Grasso, M. , Hannon, B. , Limburg, K. , Naeem, S. , O'Neill, R. V. , Paruelo, J. , Raskin, R. G. , Sutton, P. , & van den Belt, M. (1997). The value of the world's ecosystem services and natural capital. Nature, 387(6630), 253–260. 10.1038/387253a0

[gcb16495-bib-0005] Creed, I. F. , Jones, J. A. , Archer, E. , Claassen, M. , Ellison, D. , McNulty, S. G. , van Noordwijk, M. , Vira, B. , Wei, X. , Bishop, K. , Blanco, J. A. , Gush, M. , Gyawali, D. , Jobbágy, E. , Lara, A. , Little, C. , Martin‐Ortega, J. , Mukherji, A. , Murdiyarso, D. , … Xu, J. (2019). Managing forests for both downstream and downwind water. Frontiers in Forests and Global Change, 2, 64. 10.3389/FFGC.2019.00064/BIBTEX

[gcb16495-bib-0006] Curio, J. , Maussion, F. , & Scherer, D. (2015). A 12‐year high‐resolution climatology of atmospheric water transport over the Tibetan Plateau. Earth System Dynamics, 6(1), 109–124. 10.5194/esd-6-109-2015

[gcb16495-bib-0007] Dominguez, F. , Eiras‐Barca, J. , Yang, Z. , Bock, D. , Nieto, R. , & Gimeno, L. (2022). Amazonian moisture recycling revisited using WRF with water vapor tracers. Journal of Geophysical Research: Atmospheres, 127(4), e2021JD035259. 10.1029/2021JD035259

[gcb16495-bib-0008] Dominguez, F. , Hu, H. , & Martinez, J. A. (2020). Two‐layer dynamic recycling model (2L‐DRM): Learning from moisture tracking models of different complexity. Journal of Hydrometeorology, 21(1), 3–16. 10.1175/JHM-D-19-0101.1

[gcb16495-bib-0009] Fu, B. (2020). Promoting geography for sustainability. Geography and Sustainability, 1(1), 1–7. 10.1016/j.geosus.2020.02.003

[gcb16495-bib-0010] Gao, Q. Z. , Li, Y. , Wan, Y. F. , Jiangcun, W. Z. , Qin, X. B. , & Wang, B. S. (2009). Significant achievements in protection and restoration of alpine grassland ecosystem in Northern Tibet, China. Restoration Ecology, 17(3), 320–323. 10.1111/J.1526-100X.2009.00527.X

[gcb16495-bib-0011] Gao, Y. , Chen, F. , Miguez‐Macho, G. , & Li, X. (2020). Understanding precipitation recycling over the Tibetan Plateau using tracer analysis with WRF. Climate Dynamics, 55(9), 2921–2937. 10.1007/s00382-020-05426-9

[gcb16495-bib-0012] Gimeno, L. , Stohl, A. , Trigo, R. M. , Dominguez, F. , Yoshimura, K. , Yu, L. , Drumond, A. , Durán‐Quesada, A. M. , & Nieto, R. (2012). Oceanic and terrestrial sources of continental precipitation. Reviews of Geophysics, 50(4), 558–569. 10.1029/2012RG000389

[gcb16495-bib-0013] Guo, Y. , & Wang, C. (2014). Trends in precipitation recycling over the Qinghai–Xizang Plateau in last decades. Journal of Hydrology, 517, 826–835. 10.1016/J.JHYDROL.2014.06.006

[gcb16495-bib-0014] Han, C. , Ma, Y. , Wang, B. , Zhong, L. , Ma, W. , Chen, X. , & Su, Z. (2021). Long‐term variations in actual evapotranspiration over the Tibetan Plateau. Earth System Science Data, 13(7), 3513–3524. 10.5194/essd-13-3513-2021

[gcb16495-bib-0015] Hoek van Dijke, A. J. , Herold, M. , Mallick, K. , Benedict, I. , Machwitz, M. , Schlerf, M. , Schlerf, M. , Pranindita, A. , Theeuwen, J. J. E. , Bastin, J.‐F. , & Teuling, A. J. (2022). Shifts in regional water availability due to global tree restoration. Nature Geoscience, 15(5), 363–368. 10.1038/s41561-022-00935-0

[gcb16495-bib-0016] Hopping, K. A. , Knapp, A. K. , Dorji, T. , & Klein, J. A. (2018). Warming and land use change concurrently erode ecosystem services in Tibet. Global Change Biology, 24(11), 5534–5548. 10.1111/GCB.14417 30086187

[gcb16495-bib-0017] Hua, T. , Zhao, W. , Cherubini, F. , Hu, X. , & Pereira, P. (2022). Effectiveness of protected areas edges on vegetation greenness, cover and productivity on the Tibetan Plateau, China. Landscape and Urban Planning, 224, 104421. 10.1016/J.LANDURBPLAN.2022.104421

[gcb16495-bib-0018] Hua, T. , Zhao, W. , & Pereira, P. (2022). Opinionated views on grassland restoration programs on the Qinghai‐Tibetan Plateau. Frontiers in Plant Science, 13, 861200. 10.3389/FPLS.2022.861200 35557728 PMC9087572

[gcb16495-bib-0019] Huang, L. , Cao, W. , Xu, X. , Fan, J. , & Wang, J. (2018). The ecological effects of ecological security barrier protection and construction project in Tibet Plateau. Journal of Natural Resources, 33(3), 398–411. 10.11849/ZRZYXB.20170116

[gcb16495-bib-0020] Huang, L. , Shao, Q. , & Liu, J. (2019). Assessing the conservation effects of nature reserve networks under climate variability over the northeastern Tibetan Plateau. Ecological Indicators, 96, 163–173. 10.1016/J.ECOLIND.2018.08.034

[gcb16495-bib-0021] Insua‐Costa, D. , & Miguez‐Macho, G. (2018). A new moisture tagging capability in the weather research and forecasting model: Formulation, validation and application to the 2014 Great Lake‐effect snowstorm. Earth System Dynamics, 9(1), 167–185. 10.5194/esd-9-167-2018

[gcb16495-bib-0022] Jiang, W. , Lü, Y. , Liu, Y. , & Gao, W. (2020). Ecosystem service value of the Qinghai‐Tibet Plateau significantly increased during 25 years. Ecosystem Services, 44, 101146. 10.1016/J.ECOSER.2020.101146

[gcb16495-bib-0023] Jiang, Y. , Yang, K. , Shao, C. , Zhou, X. , Zhao, L. , Chen, Y. , & Wu, H. (2021). A downscaling approach for constructing high‐resolution precipitation dataset over the Tibetan Plateau from ERA5 reanalysis. Atmospheric Research, 256, 105574. 10.1016/j.atmosres.2021.105574

[gcb16495-bib-0024] Jiang, Z. , Jiang, S. , Shi, Y. , Liu, Z. , Li, W. , & Li, L. (2017). Impact of moisture source variation on decadal‐scale changes of precipitation in North China from 1951 to 2010. Journal of Geophysical Research, 122(2), 600–613. 10.1002/2016JD025795

[gcb16495-bib-0025] Keys, P. W. , Barnes, E. A. , Van Der Ent, R. J. , & Gordon, L. J. (2014). Variability of moisture recycling using a precipitationshed framework. Hydrology and Earth System Sciences, 18(10), 3937–3950. 10.5194/HESS-18-3937-2014

[gcb16495-bib-0026] Keys, P. W. , Porkka, M. , Wang‐Erlandsson, L. , Fetzer, I. , Gleeson, T. , & Gordon, L. J. (2019). Invisible water security: Moisture recycling and water resilience. Water Security, 8, 100046. 10.1016/J.WASEC.2019.100046 31875874 PMC6910651

[gcb16495-bib-0027] Keys, P. W. , & Wang‐Erlandsson, L. (2018). On the social dynamics of moisture recycling. Earth System Dynamics, 9(2), 829–847. 10.5194/esd-9-829-2018

[gcb16495-bib-0028] Keys, P. W. , Wang‐Erlandsson, L. , & Gordon, L. J. (2016). Revealing invisible water: Moisture recycling as an ecosystem service. PLoS One, 11(3), e0151993. 10.1371/journal.pone.0151993 26998832 PMC4801336

[gcb16495-bib-0029] Keys, P. W. , Wang‐Erlandsson, L. , & Gordon, L. J. (2018). Megacity precipitationsheds reveal tele‐connected water security challenges. PLoS One, 13(3), e0194311. 10.1371/JOURNAL.PONE.0194311 29534109 PMC5849328

[gcb16495-bib-0030] Keys, P. W. , Wang‐Erlandsson, L. , Gordon, L. J. , Galaz, V. , & Ebbesson, J. (2017). Approaching moisture recycling governance. Global Environmental Change, 45, 15–23. 10.1016/J.GLOENVCHA.2017.04.007

[gcb16495-bib-0031] Läderach, A. , & Sodemann, H. (2016). A revised picture of the atmospheric moisture residence time. Geophysical Research Letters, 43(2), 924–933. 10.1002/2015GL067449

[gcb16495-bib-0032] Li, C. , Zuo, Q. , Xu, X. , & Gao, S. (2016). Water vapor transport around the Tibetan Plateau and its effect on summer rainfall over the Yangtze River valley. Journal of Meteorological Research, 30(4), 472–482. 10.1007/s13351-016-5123-1

[gcb16495-bib-0033] Li, X. , Zou, L. , Xia, J. , Dou, M. , Li, H. , & Song, Z. (2022). Untangling the effects of climate change and land use/cover change on spatiotemporal variation of evapotranspiration over China. Journal of Hydrology, 612, 128189. 10.1016/j.jhydrol.2022.128189

[gcb16495-bib-0034] Lian, X. , Piao, S. , Huntingford, C. , Li, Y. , Zeng, Z. , Wang, X. , Ciais, P. , McVicar, T. R. , Peng, S. , Ottlé, C. , Yang, H. , Yang, Y. , Zhang, Y. , & Wang, T. (2018). Partitioning global land evapotranspiration using CMIP5 models constrained by observations. Nature Climate Change, 8(7), 640–646. 10.1038/s41558-018-0207-9

[gcb16495-bib-0035] Liu, W. , Mo, X. , Liu, S. , Lin, Z. , & Lv, C. (2021). Attributing the changes of grass growth, water consumed and water use efficiency over the Tibetan Plateau. Journal of Hydrology, 598, 126464. 10.1016/J.JHYDROL.2021.126464

[gcb16495-bib-0036] Liu, W. (2018). Evaluating remotely sensed monthly evapotranspiration against water balance estimates at basin scale in the Tibetan Plateau. Hydrology Research, 49(6), 1977–1990. 10.2166/NH.2018.008

[gcb16495-bib-0037] Liu, Y. (2020). The willingness to pay for ecosystem services on the Tibetan Plateau of China. Geography and Sustainability, 1(2), 141–151. 10.1016/j.geosus.2020.06.001

[gcb16495-bib-0038] Ma, N. , & Zhang, Y. (2022). Increasing Tibetan Plateau terrestrial evapotranspiration primarily driven by precipitation. Agricultural and Forest Meteorology, 317, 108887. 10.1016/J.AGRFORMET.2022.108887

[gcb16495-bib-0039] Martens, B. , Miralles, D. G. , Lievens, H. , van der Schalie, R. , de Jeu, R. A. M. , Fernández‐Prieto, D. , Beck, H. E. , Dorigo, W. A. , & Verhoest, N. E. C. (2017). GLEAM v3: Satellite‐based land evaporation and root‐zone soil moisture. Geoscientific Model Development, 10(5), 1903–1925. 10.5194/gmd-10-1903-2017

[gcb16495-bib-0040] Miralles, D. G. , Nieto, R. , McDowell, N. G. , Dorigo, W. A. , Verhoest, N. E. , Liu, Y. Y. , Teuling, A. J. , Dolman, A. J. , Good, S. P. , & Gimeno, L. (2016). Contribution of water‐limited ecoregions to their own supply of rainfall. Environmental Research Letters, 11(12), 124007. 10.1088/1748-9326/11/12/124007

[gcb16495-bib-0041] Mu, Y. , Biggs, T. W. , & De Sales, F. (2021). Forests mitigate drought in an agricultural region of the Brazilian Amazon: Atmospheric moisture tracking to identify critical source areas. Geophysical Research Letters, 48(5), e2020GL091380. 10.1029/2020GL091380

[gcb16495-bib-0042] O'Connor, J. C. , Dekker, S. C. , Staal, A. , Tuinenburg, O. A. , Rebel, K. T. , & Santos, M. J. (2021). Forests buffer against variations in precipitation. Global Change Biology, 27(19), 4686–4696. 10.1111/GCB.15763 34319636 PMC8457185

[gcb16495-bib-0043] Pranindita, A. , Wang‐Erlandsson, L. , Fetzer, I. , & Teuling, A. J. (2022). Moisture recycling and the potential role of forests as moisture source during European heatwaves. Climate Dynamics, 58(1–2), 609–624. 10.1007/s00382-021-05921-7 35125663 PMC8791891

[gcb16495-bib-0044] Shen, M. , Piao, S. , Jeong, S.‐J. , Zhou, L. , Zeng, Z. , Ciais, P. , Chen, D. , Huang, M. , Jin, C. S. , Li, L. Z. , Li, Y. , Myneni, R. B. , Yang, K. , Zhang, G. , Zhang, Y. , & Yao, T. (2015). Evaporative cooling over the Tibetan Plateau induced by vegetation growth. Proceedings of the National Academy of Sciences of the United States of America, 112(30), 9299–9304. 10.1073/pnas.1504418112 26170316 PMC4522821

[gcb16495-bib-0045] Shen, M. , Wang, S. , Jiang, N. , Sun, J. , Cao, R. , Ling, X. , Ling, X. , Fang, B. , Zhang, L. , Zhang, L. , Xu, X. , Lv, W. , Li, B. , Sun, Q. , Meng, F. , Jiang, Y. , Dorji, T. , Fu, Y. , Iler, A. , … Fu, B. (2022). Plant phenology changes and drivers on the Qinghai–Tibetan Plateau. Nature Reviews Earth & Environment, 3, 633–651. 10.1038/s43017-022-00317-5

[gcb16495-bib-0046] Shen, Y. , Liu, G. , Zhou, W. , Liu, Y. , Cheng, H. , & Su, X. (2021). Protected areas have remarkable spillover effects on forest conservation on the Qinghai‐Tibet Plateau. Diversity and Distributions, 1–12. 10.1111/ddi.13466

[gcb16495-bib-0047] Snyder, P. K. , Delire, C. , & Foley, J. A. (2004). Evaluating the influence of different vegetation biomes on the global climate. Climate Dynamics, 23(3–4), 279–302. 10.1007/s00382-004-0430-0

[gcb16495-bib-0048] Song, Z. , Feng, Q. , Gao, Z. , Cao, S. , Cao, G. , & Wang, Z. (2022). Temporal and spatial differences and driving factors of evapotranspiration from terrestrial ecosystems of the Qinghai Province in the past 20 years. Water, 14(4), 536. 10.3390/w14040536

[gcb16495-bib-0049] Staal, A. , Tuinenburg, O. A. , Bosmans, J. H. C. , Holmgren, M. , van Nes, E. H. , Scheffer, M. , Zemp, D. C. , & Dekker, S. C. (2018). Forest‐rainfall cascades buffer against drought across the Amazon. Nature Climate Change, 8(6), 539–543. 10.1038/s41558-018-0177-y

[gcb16495-bib-0050] Sun, H. , Zheng, D. , Yao, T. , & Zhang, Y. (2012). Protection and construction of the national ecological security shelter zone on Tibetan Plateau. Acta Geographica Sinica, 67(1), 3–12. http://www.geog.com.cn

[gcb16495-bib-0051] te Wierik, S. A. , Cammeraat, E. L. H. , Gupta, J. , & Artzy‐Randrup, Y. A. (2021). Reviewing the impact of land use and land‐use change on moisture recycling and precipitation patterns. Water Resources Research, 57(7), e2020WR029234. 10.1029/2020WR029234

[gcb16495-bib-0052] Tuinenburg, O. A. , Bosmans, J. H. C. , & Staal, A. (2022). The global potential of forest restoration for drought mitigation. Environmental Research Letters, 17(3), 034045. 10.1088/1748-9326/AC55B8

[gcb16495-bib-0053] Tuinenburg, O. A. , & Staal, A. (2020). Tracking the global flows of atmospheric moisture and associated uncertainties. Hydrology and Earth System Sciences, 24(5), 2419–2435. 10.5194/HESS-24-2419-2020

[gcb16495-bib-0054] Tuinenburg, O. A. , Theeuwen, J. J. E. , & Staal, A. (2020). High‐resolution global atmospheric moisture connections from evaporation to precipitation. Earth System Science Data, 12(4), 3177–3188. 10.5194/essd-12-3177-2020

[gcb16495-bib-0055] Van Der Ent, R. J. , Wang‐Erlandsson, L. , Keys, P. W. , & Savenije, H. H. G. (2014). Contrasting roles of interception and transpiration in the hydrological cycle part 2: Moisture recycling. Earth System Dynamics, 5(2), 471–489. 10.5194/ESD-5-471-2014

[gcb16495-bib-0056] van der Ent, R. J. , Savenije, H. H. G. , Schaefli, B. , & Steele‐Dunne, S. C. (2010). Origin and fate of atmospheric moisture over continents. Water Resources Research, 46(9), 1–12. 10.1029/2010WR009127

[gcb16495-bib-0057] Wang, T. , Yang, D. , Yang, Y. , Piao, S. , Li, X. , Cheng, G. , & Fu, B. (2020). Permafrost thawing puts the frozen carbon at risk over the Tibetan Plateau. Science Advances, 6(19), 2–10. 10.1126/sciadv.aaz3513 PMC7202872 32494710

[gcb16495-bib-0058] Wang, W. , Li, J. , Yu, Z. , Ding, Y. , Xing, W. , & Lu, W. (2018). Satellite retrieval of actual evapotranspiration in the Tibetan Plateau: Components partitioning, multidecadal trends and dominated factors identifying. Journal of Hydrology, 559, 471–485. 10.1016/J.JHYDROL.2018.02.065

[gcb16495-bib-0059] Wang, X. , Cheng, G. , Zhao, T. , Zhang, X. , Zhu, L. , & Huang, L. (2017). Assessment on protection and construction of ecological safety shelter for Tibet. Bulletin of Chinese Academy of Sciences, 32(1), 29–34. 10.16418/j.issn.1000-3045.2017.01.004 (Chinese Version).

[gcb16495-bib-0060] Wang‐Erlandsson, L. , Van Der Ent, R. J. , Gordon, L. J. , & Savenije, H. H. G. (2014). Contrasting roles of interception and transpiration in the hydrological cycle ‐ Part 1: Temporal characteristics over land. Earth System Dynamics, 5(2), 441–469. 10.5194/ESD-5-441-2014

[gcb16495-bib-0061] Wang‐Erlandsson, L. , Fetzer, I. , Keys, P. W. , Van Der Ent, R. J. , Savenije, H. H. G. , & Gordon, L. J. (2018). Remote land use impacts on river flows through atmospheric teleconnections. Hydrology and Earth System Sciences, 22(8), 4311–4328. 10.5194/HESS-22-4311-2018

[gcb16495-bib-0062] Wei, D. (2019). Geographical distribution of major ecological projects on the Tibetan Plateau. National Tibetan Plateau Data Center.

[gcb16495-bib-0063] Wei, D. , Qi, Y. , Ma, Y. , Wang, X. , Ma, W. , Gao, T. , Huang, L. , Zhao, H. , Zhang, J. , & Wang, X. (2021). Plant uptake of CO_2_ outpaces losses from permafrost and plant respiration on the Tibetan Plateau. Proceedings of the National Academy of Sciences of the United States of America, 118(33), e2015283118. 10.1073/pnas.2015283118 34373324 PMC8379928

[gcb16495-bib-0064] Wei, J. , & Dirmeyer, P. A. (2019). Sensitivity of land precipitation to surface evapotranspiration: A nonlocal perspective based on water vapor transport. Geophysical Research Letters, 46(21), 12588–12597. 10.1029/2019GL085613

[gcb16495-bib-0065] Wei, J. , Dirmeyer, P. A. , Bosilovich, M. G. , & Wu, R. (2012). Water vapor sources for Yangtze River valley rainfall: Climatology, variability, and implications for rainfall forecasting. Journal of Geophysical Research: Atmospheres, 117(D5), 5126. 10.1029/2011JD016902

[gcb16495-bib-0066] Wei, J. , Jin, Q. , Yang, Z. L. , & Dirmeyer, P. A. (2016). Role of ocean evaporation in California droughts and floods. Geophysical Research Letters, 43(12), 6554–6562. 10.1002/2016GL069386

[gcb16495-bib-0067] Wei, Z. , Yoshimura, K. , Wang, L. , Miralles, D. G. , Jasechko, S. , & Lee, X. (2017). Revisiting the contribution of transpiration to global terrestrial evapotranspiration. Geophysical Research Letters, 44(6), 2792–2801. 10.1002/2016GL072235

[gcb16495-bib-0068] Windisch, M. G. , Davin, E. L. , & Seneviratne, S. I. (2021). Prioritizing forestation based on biogeochemical and local biogeophysical impacts. Nature Climate Change, 11(10), 867–871. 10.1038/s41558-021-01161-z

[gcb16495-bib-0069] Worden, S. , Fu, R. , Chakraborty, S. , Liu, J. , & Worden, J. (2021). Where does moisture come from over the Congo Basin? Journal of Geophysical Research Biogeosciences, 126(8), e2020JG006024. 10.1029/2020JG006024

[gcb16495-bib-0070] Xiao, Y. , Xiong, Q. , Liang, P. , & Xiao, Q. (2021). Potential risk to water resources under eco‐restoration policy and global change in the Tibetan Plateau. Environmental Research Letters, 16(9), 094004. 10.1088/1748-9326/AC1819

[gcb16495-bib-0071] Xu, Y. , & Gao, Y. (2019). Quantification of evaporative sources of precipitation and its changes in the Southeastern Tibetan Plateau and Middle Yangtze River Basin. Atmosphere, 10(8), 428. 10.3390/atmos10080428

[gcb16495-bib-0072] Yang, H. , Shen, X. , Yao, J. , & Wen, Q. (2020). Portraying the impact of the Tibetan Plateau on global climate. Journal of Climate, 33(9), 3565–3583. 10.1175/JCLI-D-18-0734.1

[gcb16495-bib-0073] Yang, K. , Tang, Q. , & Lu, H. (2022). Precipitation recycling ratio and water vapor sources on the Tibetan Plateau. Science China Earth Sciences, 65(3), 584–588. 10.1007/s11430-021-9871-5

[gcb16495-bib-0074] Yang, Z. , Qian, Y. , Liu, Y. , Berg, L. K. , Hu, H. , Dominguez, F. , Yang, B. , Feng, Z. , Gustafson, W. I., Jr. , Huang, M. , & Tang, Q. (2019). Irrigation impact on water and energy cycle during dry years over the United States using convection‐permitting WRF and a dynamical recycling model. Journal of Geophysical Research: Atmospheres, 124(21), 11220–11241. 10.1029/2019JD030524

[gcb16495-bib-0075] Yu, D. , Li, X. , Cao, Q. , Hao, R. , & Qiao, J. (2020). Impacts of climate variability and landscape pattern change on evapotranspiration in a grassland landscape mosaic. Hydrological Processes, 34(4), 1035–1051. 10.1002/HYP.13642

[gcb16495-bib-0076] Yuan, X. , Yang, K. , Lu, H. , He, J. , Sun, J. , & Wang, Y. (2021). Characterizing the features of precipitation for the Tibetan Plateau among four gridded datasets: Detection accuracy and spatio‐temporal variabilities. Atmospheric Research, 264, 105875. 10.1016/j.atmosres.2021.105875

[gcb16495-bib-0077] Zemp, D. C. , Schleussner, C.‐F. , Barbosa, H. M. J. , van der Ent, R. J. , Donges, J. F. , Heinke, J. , Sampaio, G. , & Rammig, A. (2014). On the importance of cascading moisture recycling in South America. Atmospheric Chemistry and Physics, 14(23), 13337–13359. 10.5194/acp-14-13337-2014

[gcb16495-bib-0078] Zhang, C. , Tang, Q. , & Chen, D. (2017). Recent changes in the moisture source of precipitation over the Tibetan Plateau. Journal of Climate, 30(5), 1821–1837. 10.1175/JCLI-D-16-0493.1

[gcb16495-bib-0079] Zhang, Y. (2020). PML_V2 global evapotranspiration and gross primary production (2002.07‐2019.08). National Tibetan Plateau Data Center. 10.11888/Geogra.tpdc.270251

[gcb16495-bib-0080] Zhang, Y. , Kong, D. , Gan, R. , Chiew, F. H. S. , McVicar, T. R. , Zhang, Q. , & Yang, Y. (2019). Coupled estimation of 500 m and 8‐day resolution global evapotranspiration and gross primary production in 2002–2017. Remote Sensing of Environment, 222, 165–182. 10.1016/J.RSE.2018.12.031

[gcb16495-bib-0081] Zhang, Y. , Peña‐Arancibia, J. L. , McVicar, T. R. , Chiew, F. H. S. , Vaze, J. , Liu, C. , Lu, X. , Zheng, H. , Wang, Y. , Liu, Y. Y. , Miralles, D. G. , & Pan, M. (2016). Multi‐decadal trends in global terrestrial evapotranspiration and its components. Scientific Reports, 6(1), 1–12. 10.1038/srep19124 26750505 PMC4707530

[gcb16495-bib-0082] Zhao, D. , Zhu, Y. , Wu, S. , & Zheng, D. (2021). Projection of vegetation distribution to 1.5°C and 2°C of global warming on the Tibetan Plateau. Global and Planetary Change, 202, 103525. 10.1016/J.GLOPLACHA.2021.103525

[gcb16495-bib-0083] Zhao, Y. , & Zhou, T. (2021). Interannual variability of precipitation recycle ratio over the Tibetan Plateau. Journal of Geophysical Research: Atmospheres, 126(2), e2020JD033733. 10.1029/2020JD033733

[gcb16495-bib-0084] Zheng, H. , Miao, C. , Li, X. , Kong, D. , Gou, J. , Wu, J. , & Zhang, S. (2022). Effects of vegetation changes and multiple environmental factors on evapotranspiration across China over the past 34 years. Earth's Futures, 10(4), e2021EF002564. 10.1029/2021EF002564

