# Supplementary material for: Contribution of Tibetan Plateau ecosystems to local and remote precipitation through moisture recycling
Source: Glob Chang Biol. 2022 Nov 11;29(3):702–18. doi: 10.1111/gcb.16495 (PMC10099335; doi:10.1111/gcb.16495)
Supplement: Supplementary file 1 — Appendix S1 [file GCB-29-702-s001.docx]

Table S1. Precipitation contribution of different ecosystems and ecological project areas in TP to different regions through ET (mm/year (%)). Numbers in parentheses denote relative contribution.

|  | **Precipitation contribution by ecosystem types (mm/yr (%))** | | | | | **Precipitation contribution by ecological project areas (mm/yr (%))** | | | |
| --- | --- | --- | --- | --- | --- | --- | --- | --- | --- |
| **Region** | Forest | Shrub | Grass | Barren/snow | Other | Grassland protection | Forestland protection | Erosion control | Desertification control |
| Qinghai | 3.8 (0.73%) | 2.6 (0.5%) | 223 (43%) | 80 (15%) | 3.4 (0.65%) | 241 (46%) | 93 (18%) | 149 (29%) | 112 (22%) |
| Sichuan | 46 (3.6%) | 33 (2.6%) | 192 (15%) | 15 (1.2%) | 1.7 (0.13%) | 222 (17%) | 253 (20%) | 135 (11%) | 45 (3.5%) |
| Xizang | 4.8 (0.55%) | 0.93 (0.11%) | 106 (12%) | 77 (8.9%) | 0.18 (0.02%) | 161 (19%) | 42 (4.8%) | 41 (4.8%) | 65 (7.5%) |
| Gansu | 8.7 (2%) | 6.1 (1.4%) | 91 (21%) | 23 (5.3%) | 5.6 (1.3%) | 87 (20%) | 71 (17%) | 51 (12%) | 38 (8.8%) |
| Chongqing | 20.0 (1.5%) | 16 (1.2%) | 34 (2.5%) | 3.1 (0.23%) | 0.97 (0.07%) | 53 (3.8%) | 67 (4.9%) | 30.0 (2.1%) | 7.9 (0.57%) |
| Shaanxi | 9.3 (1.1%) | 6.2 (0.75%) | 40 (4.8%) | 6.5 (0.78%) | 2.8 (0.34%) | 47 (5.6%) | 43 (5.2%) | 23 (2.7%) | 16 (1.9%) |
| Ningxia | 4.3 (1.1%) | 3 (0.79%) | 41 (11%) | 9.3 (2.4%) | 4.2 (1.1%) | 38 (10%) | 31 (8.3%) | 23 (6%) | 17 (4.6%) |
| Guizhou | 11 (0.71%) | 8.9 (0.58%) | 12 (0.81%) | 1.2 (0.08%) | 0.28 (0.02%) | 18 (1.2%) | 31 (2%) | 11 (0.72%) | 2.5 (0.17%) |
| Hubei | 7.2 (0.54%) | 5.5 (0.41%) | 16 (1.2%) | 2.3 (0.17%) | 0.71 (0.05%) | 22 (1.6%) | 25 (1.9%) | 12 (0.89%) | 4.8 (0.36%) |
| Shanxi | 2.4 (0.39%) | 1.6 (0.27%) | 17 (2.8%) | 4.6 (0.76%) | 1.5 (0.25%) | 17 (2.8%) | 15 (2.4%) | 10 (1.7%) | 7.2 (1.2%) |
| Yunnan | 8.7 (0.54%) | 6.3 (0.39%) | 7.6 (0.47%) | 0.92 (0.06%) | 0.09 (0.01%) | 8.3 (0.52%) | 23 (1.4%) | 5.9 (0.37%) | 0.9 (0.06%) |
| Xinjiang | 0.0 (0%) | 0.01 (0%) | 3.5 (1.4%) | 15 (5.9%) | 0.48 (0.19%) | 5.5 (2.2%) | 0.21 (0.08%) | 1.2 (0.47%) | 1.3 (0.53%) |
| Henan | 2.5 (0.33%) | 1.8 (0.24%) | 9.6 (1.3%) | 2.1 (0.27%) | 0.6 (0.08%) | 11 (1.5%) | 11 (1.4%) | 6.1 (0.8%) | 3.6 (0.48%) |
| Hunan | 4.3 (0.27%) | 3.5 (0.21%) | 7.4 (0.46%) | 1 (0.06%) | 0.28 (0.02%) | 10 (0.63%) | 14 (0.86%) | 5.9 (0.36%) | 2.1 (0.13%) |
| Anhui | 2.6 (0.2%) | 2 (0.15%) | 7 (0.54%) | 1.4 (0.11%) | 0.33 (0.03%) | 8.7 (0.67%) | 9.6 (0.74%) | 4.9 (0.38%) | 2.3 (0.18%) |
| Zhejiang | 2.2 (0.13%) | 1.7 (0.1%) | 5.2 (0.3%) | 0.99 (0.06%) | 0.24 (0.01%) | 6.6 (0.38%) | 7.8 (0.46%) | 3.8 (0.22%) | 1.7 (0.1%) |
| Hebei | 0.75 (0.13%) | 0.54 (0.1%) | 6.1 (1.1%) | 2.2 (0.38%) | 0.6 (0.11%) | 6 (1.1%) | 5.4 (0.95%) | 4 (0.7%) | 2.8 (0.49%) |
| Jiangxi | 2.5 (0.14%) | 1.9 (0.11%) | 4.8 (0.27%) | 0.76 (0.04%) | 0.19 (0.01%) | 6.3 (0.35%) | 8.2 (0.46%) | 3.6 (0.2%) | 1.4 (0.08%) |
| Beijing | 0.64 (0.11%) | 0.44 (0.07%) | 5.6 (0.94%) | 2.2 (0.37%) | 0.56 (0.09%) | 5.6 (0.94%) | 4.9 (0.82%) | 3.8 (0.63%) | 2.6 (0.44%) |
| Jiangsu | 1.6 (0.14%) | 1.2 (0.11%) | 5 (0.45%) | 1.1 (0.1%) | 0.27 (0.02%) | 6 (0.55%) | 6.2 (0.57%) | 3.4 (0.31%) | 1.8 (0.16%) |
| Shanghai | 1.7 (0.13%) | 1.2 (0.09%) | 4.8 (0.36%) | 1.1 (0.08%) | 0.23 (0.02%) | 5.8 (0.44%) | 6.4 (0.48%) | 3.3 (0.25%) | 1.6 (0.12%) |
| Shandong | 0.97 (0.14%) | 0.7 (0.1%) | 4.7 (0.69%) | 1.3 (0.19%) | 0.37 (0.05%) | 5.1 (0.76%) | 4.8 (0.71%) | 3 (0.45%) | 1.9 (0.28%) |
| Tianjin | 0.62 (0.1%) | 0.46 (0.08%) | 4.7 (0.77%) | 1.5 (0.25%) | 0.46 (0.08%) | 4.7 (0.78%) | 4.3 (0.71%) | 3.1 (0.51%) | 2.2 (0.36%) |
| Neimeng | 0.35 (0.1%) | 0.25 (0.07%) | 4.7 (1.4%) | 1.7 (0.52%) | 0.52 (0.16%) | 4.1 (1.2%) | 3.7 (1.1%) | 3.2 (0.96%) | 2.2 (0.67%) |
| Liaoning | 0.52 (0.07%) | 0.38 (0.05%) | 4.3 (0.61%) | 1.7 (0.24%) | 0.43 (0.06%) | 4.3 (0.6%) | 3.8 (0.53%) | 2.9 (0.41%) | 2 (0.28%) |
| Taiwan | 1.4 (0.05%) | 1.1 (0.04%) | 3.1 (0.11%) | 0.76 (0.03%) | 0.15 (0%) | 4 (0.14%) | 4.6 (0.16%) | 2.4 (0.08%) | 1.1 (0.04%) |
| Jilin | 0.32 (0.04%) | 0.23 (0.03%) | 3.1 (0.42%) | 1.3 (0.18%) | 0.32 (0.04%) | 3 (0.4%) | 2.6 (0.35%) | 2.1 (0.29%) | 1.5 (0.2%) |
| Fujian | 1.1 (0.06%) | 0.86 (0.05%) | 2.2 (0.12%) | 0.4 (0.02%) | 0.09 (0.01%) | 2.8 (0.16%) | 3.6 (0.21%) | 1.7 (0.09%) | 0.68 (0.04%) |
| Guangxi | 1 (0.06%) | 0.79 (0.04%) | 1.7 (0.09%) | 0.28 (0.01%) | 0.07 (0%) | 2.2 (0.11%) | 3.2 (0.17%) | 1.3 (0.07%) | 0.49 (0.03%) |
| Heilongjiang | 0.12 (0.02%) | 0.08 (0.01%) | 1.3 (0.2%) | 0.58 (0.09%) | 0.15 (0.02%) | 1.2 (0.19%) | 1.1 (0.17%) | 0.94 (0.14%) | 0.66 (0.1%) |
| Guangdong | 0.5 (0.03%) | 0.39 (0.02%) | 0.91 (0.05%) | 0.17 (0.01%) | 0.04 (0%) | 1.2 (0.06%) | 1.6 (0.09%) | 0.71 (0.04%) | 0.28 (0.02%) |
| Hongkong | 0.29 (nan) | 0.24 (nan) | 0.59 (nan) | 0.11 (nan) | 0.03 (nan) | 0.76 (nan) | 0.98 (nan) | 0.51 (nan) | 0.17 (nan) |
| Hainan | 0.28 (0.02%) | 0.23 (0.01%) | 0.6 (0.03%) | 0.11 (0.01%) | 0.04 (0%) | 0.74 (0.04%) | 0.96 (0.05%) | 0.44 (0.02%) | 0.18 (0.01%) |
| TP | 9.1  (1.2%) | 5.2  (0.68%) | 138  (18%) | 65  (8.4%) | 1.7  (0.22%) | 169  (22%) | 82  (11%) | 79  (10%) | 66  (8.6) |

Table S2. Precipitation contribution of different ecosystems and ecological project areas in TP to different regions through T (mm/year (%)). Numbers in parentheses denote relative contribution.

|  | **Precipitation contribution by**  **ecosystem types (mm/yr (%))** | | | | | **Precipitation contribution by**  **ecological project areas (mm/yr (%))** | | | |
| --- | --- | --- | --- | --- | --- | --- | --- | --- | --- |
| **Region** | Forest | Shrub | Grass | Barren/snow | Other | Grassland protection | Forestland protection | Erosion control | Desertification control |
| Sichuan | 34 (2.7%) | 25 (2%) | 152 (12%) | 8.1 (0.63%) | 1.4 (0.11%) | 174 (14%) | 201 (16%) | 106 (8.3%) | 33 (2.6%) |
| Qinghai | 2.8 (0.54%) | 2 (0.38%) | 129 (25%) | 15 (3%) | 2.5 (0.48%) | 119 (23%) | 68 (13%) | 88 (17%) | 52 (10%) |
| Gansu | 6.5 (1.5%) | 4.7 (1.1%) | 66 (15%) | 5.1 (1.2%) | 4.2 (0.98%) | 61 (14%) | 53 (12%) | 33 (7.7%) | 25 (5.8%) |
| Xizang | 3.3 (0.38%) | 0.43 (0.05%) | 43 (4.9%) | 14 (1.6%) | 0.11 (0.01%) | 46 (5.3%) | 28 (3.2%) | 24 (2.8%) | 23 (2.7%) |
| Chongqing | 16 (1.1%) | 13 (0.93%) | 27 (2%) | 1.4 (0.1%) | 0.76 (0.06%) | 41 (2.9%) | 53 (3.8%) | 23 (1.7%) | 5.8 (0.42%) |
| Shaanxi | 6.9 (0.83%) | 4.8 (0.58%) | 30 (3.6%) | 1.7 (0.21%) | 2.1 (0.26%) | 34 (4.1%) | 33 (4%) | 16 (1.9%) | 11 (1.3%) |
| Ningxia | 3.1 (0.82%) | 2.3 (0.61%) | 30.0 (7.8%) | 2.2 (0.57%) | 3.1 (0.82%) | 26 (6.8%) | 23 (6%) | 14 (3.8%) | 11 (2.9%) |
| Guizhou | 8.1 (0.53%) | 7 (0.46%) | 9.6 (0.63%) | 0.62 (0.04%) | 0.22 (0.01%) | 14 (0.9%) | 24 (1.6%) | 8.6 (0.57%) | 1.8 (0.12%) |
| Hubei | 5.4 (0.41%) | 4.4 (0.33%) | 12 (0.93%) | 0.74 (0.06%) | 0.55 (0.04%) | 16 (1.2%) | 20.0 (1.5%) | 8.9 (0.67%) | 3.3 (0.25%) |
| Yunnan | 6.2 (0.39%) | 4.8 (0.3%) | 5.8 (0.36%) | 0.6 (0.04%) | 0.07 (0%) | 6.3 (0.39%) | 17 (1.1%) | 4.5 (0.28%) | 0.65 (0.04%) |
| Shanxi | 1.7 (0.29%) | 1.3 (0.21%) | 12 (2%) | 1.1 (0.18%) | 1.1 (0.18%) | 11 (1.9%) | 10 (1.7%) | 6.3 (1%) | 4.4 (0.73%) |
| Hunan | 3.3 (0.2%) | 2.8 (0.17%) | 5.7 (0.35%) | 0.37 (0.02%) | 0.21 (0.01%) | 7.7 (0.47%) | 11 (0.67%) | 4.5 (0.27%) | 1.4 (0.09%) |
| Henan | 1.9 (0.25%) | 1.4 (0.19%) | 7.1 (0.93%) | 0.53 (0.07%) | 0.45 (0.06%) | 7.9 (1%) | 8.3 (1.1%) | 4.3 (0.56%) | 2.3 (0.3%) |
| Anhui | 2 (0.15%) | 1.6 (0.12%) | 5.2 (0.4%) | 0.38 (0.03%) | 0.25 (0.02%) | 6.3 (0.48%) | 7.5 (0.58%) | 3.6 (0.28%) | 1.5 (0.12%) |
| Jiangxi | 1.9 (0.11%) | 1.5 (0.09%) | 3.6 (0.2%) | 0.25 (0.01%) | 0.15 (0.01%) | 4.7 (0.26%) | 6.4 (0.36%) | 2.7 (0.15%) | 0.95 (0.05%) |
| Zhejiang | 1.7 (0.1%) | 1.4 (0.08%) | 3.9 (0.23%) | 0.28 (0.02%) | 0.18 (0.01%) | 4.8 (0.28%) | 6.1 (0.36%) | 2.8 (0.16%) | 1.1 (0.06%) |
| Shanghai | 1.3 (0.1%) | 1 (0.07%) | 3.6 (0.27%) | 0.28 (0.02%) | 0.17 (0.01%) | 4.2 (0.31%) | 5 (0.37%) | 2.4 (0.18%) | 1 (0.08%) |
| Jiangsu | 1.2 (0.11%) | 0.95 (0.09%) | 3.7 (0.33%) | 0.29 (0.03%) | 0.2 (0.02%) | 4.2 (0.39%) | 4.8 (0.44%) | 2.4 (0.22%) | 1.1 (0.1%) |
| Hebei | 0.55 (0.1%) | 0.42 (0.07%) | 4.3 (0.76%) | 0.51 (0.09%) | 0.42 (0.07%) | 3.9 (0.68%) | 3.6 (0.64%) | 2.3 (0.41%) | 1.6 (0.28%) |
| Beijing | 0.47 (0.08%) | 0.34 (0.06%) | 3.9 (0.66%) | 0.51 (0.09%) | 0.38 (0.06%) | 3.5 (0.59%) | 3.2 (0.54%) | 2.2 (0.36%) | 1.5 (0.25%) |
| Shandong | 0.73 (0.11%) | 0.55 (0.08%) | 3.4 (0.5%) | 0.31 (0.05%) | 0.27 (0.04%) | 3.5 (0.51%) | 3.5 (0.52%) | 2 (0.29%) | 1.2 (0.17%) |
| Xinjiang | 0.0 (0%) | 0.0 (0%) | 1.5 (0.6%) | 3.1 (1.2%) | 0.31 (0.12%) | 0.91 (0.36%) | 0.09 (0.04%) | 0.32 (0.13%) | 0.31 (0.12%) |
| Tianjin | 0.46 (0.08%) | 0.35 (0.06%) | 3.3 (0.54%) | 0.37 (0.06%) | 0.32 (0.05%) | 3.1 (0.51%) | 2.9 (0.48%) | 1.8 (0.3%) | 1.3 (0.21%) |
| Taiwan | 1 (0.04%) | 0.85 (0.03%) | 2.3 (0.08%) | 0.2 (0.01%) | 0.11 (0%) | 2.8 (0.1%) | 3.6 (0.12%) | 1.8 (0.06%) | 0.64 (0.02%) |
| Neimeng | 0.25 (0.08%) | 0.19 (0.06%) | 3.2 (0.97%) | 0.42 (0.13%) | 0.35 (0.11%) | 2.6 (0.77%) | 2.3 (0.7%) | 1.8 (0.52%) | 1.3 (0.38%) |
| Liaoning | 0.38 (0.05%) | 0.29 (0.04%) | 3 (0.43%) | 0.39 (0.05%) | 0.3 (0.04%) | 2.7 (0.39%) | 2.5 (0.36%) | 1.7 (0.24%) | 1.1 (0.16%) |
| Fujian | 0.86 (0.05%) | 0.69 (0.04%) | 1.6 (0.09%) | 0.12 (0.01%) | 0.07 (0%) | 2 (0.12%) | 2.8 (0.16%) | 1.2 (0.07%) | 0.43 (0.02%) |
| Jilin | 0.24 (0.03%) | 0.18 (0.02%) | 2.2 (0.29%) | 0.31 (0.04%) | 0.22 (0.03%) | 1.9 (0.25%) | 1.7 (0.23%) | 1.2 (0.16%) | 0.82 (0.11%) |
| Guangxi | 0.79 (0.04%) | 0.62 (0.03%) | 1.3 (0.07%) | 0.1 (0.01%) | 0.05 (0%) | 1.6 (0.08%) | 2.5 (0.13%) | 0.96 (0.05%) | 0.32 (0.02%) |
| Guangdong | 0.38 (0.02%) | 0.31 (0.02%) | 0.68 (0.04%) | 0.06 (0%) | 0.03 (0%) | 0.85 (0.05%) | 1.2 (0.07%) | 0.53 (0.03%) | 0.18 (0.01%) |
| Heilongjiang | 0.09 (0.01%) | 0.07 (0.01%) | 0.93 (0.14%) | 0.14 (0.02%) | 0.1 (0.02%) | 0.76 (0.12%) | 0.71 (0.11%) | 0.51 (0.08%) | 0.36 (0.06%) |
| Hainan | 0.21 (0.01%) | 0.18 (0.01%) | 0.46 (0.03%) | 0.03 (0%) | 0.03 (0%) | 0.54 (0.03%) | 0.73 (0.04%) | 0.32 (0.02%) | 0.12 (0.01%) |
| Hongkong | 0.22 (nan) | 0.19 (nan) | 0.43 (nan) | 0.03 (nan) | 0.02 (nan) | 0.54 (nan) | 0.75 (nan) | 0.37 (nan) | 0.11 (nan) |
| TP | 6.7 (0.87%) | 3.9 (0.51%) | 79 (10%) | 13 (1.7%) | 1.2 (0.16%) | 79 (10%) | 61 (8%) | 51 (6.6%) | 30 (3.9%) |

Table S3. Precipitation contribution of TP ecosystems through ET in different seasons (mm/season (%)). Numbers in parentheses denote relative contribution.

| **Region** | MAM | JJA | SON | DJF | Annual |
| --- | --- | --- | --- | --- | --- |
| Qinghai | 59 (61%) | 179 (63%) | 66 (58%) | 12 (51%) | 316 (61%) |
| Sichuan | 66 (25%) | 142 (23%) | 68 (21%) | 13 (16%) | 288 (23%) |
| Xizang | 28 (16%) | 124 (26%) | 36 (23%) | 3.8 (6.1%) | 192 (22%) |
| Gansu | 37 (41%) | 56 (27%) | 34 (30%) | 8 (34%) | 135 (31%) |
| Chongqing | 33 (8.3%) | 20 (3.8%) | 14 (3.8%) | 9.2 (8%) | 75 (5.4%) |
| Shaanxi | 26 (16%) | 14 (3.7%) | 18 (7.4%) | 7.3 (16%) | 65 (7.8%) |
| Ningxia | 17 (24%) | 18 (11%) | 22 (18%) | 4.8 (28%) | 62 (16%) |
| Guizhou | 14 (3.1%) | 9.8 (1.7%) | 5.1 (1.6%) | 4.4 (2.9%) | 34 (2.2%) |
| Hubei | 15 (4%) | 6.8 (1.2%) | 5.5 (1.9%) | 4.9 (4.5%) | 32 (2.4%) |
| Shanxi | 9.4 (9.4%) | 6.2 (1.9%) | 8.7 (5.5%) | 2.7 (12%) | 27 (4.5%) |
| Yunnan | 4.7 (1.6%) | 13 (1.5%) | 4.8 (1.4%) | 1.3 (1.2%) | 24 (1.5%) |
| Xinjiang | 4.1 (6.5%) | 10 (9.1%) | 3.6 (7%) | 0.86 (3.7%) | 19 (7.5%) |
| Henan | 6.6 (4.4%) | 3 (0.79%) | 4.3 (2.3%) | 2.8 (6.6%) | 17 (2.2%) |
| Hunan | 8.6 (1.4%) | 3.1 (0.55%) | 2.1 (0.76%) | 2.8 (1.5%) | 17 (1%) |
| Anhui | 5.8 (1.8%) | 2 (0.33%) | 2.1 (0.92%) | 3.3 (2.4%) | 13 (1%) |
| Zhejiang | 5.1 (1%) | 1.1 (0.15%) | 1.4 (0.45%) | 2.8 (1.3%) | 10 (0.61%) |
| Hebei | 2.9 (3.6%) | 3 (0.85%) | 3.3 (2.8%) | 0.99 (6.6%) | 10 (1.8%) |
| Jiangxi | 5.4 (0.81%) | 1.3 (0.2%) | 1.3 (0.48%) | 2.2 (1%) | 10 (0.57%) |
| Beijing | 2.4 (3.3%) | 3 (0.78%) | 3.3 (2.5%) | 0.86 (6.1%) | 9.5 (1.6%) |
| Jiangsu | 3.4 (1.6%) | 1.6 (0.29%) | 1.7 (0.75%) | 2.4 (2.4%) | 9.2 (0.83%) |
| Shanghai | 3.9 (1.2%) | 1.2 (0.2%) | 1.3 (0.51%) | 2.7 (1.6%) | 9.1 (0.68%) |
| Shandong | 2.8 (2.4%) | 1.8 (0.45%) | 1.9 (1.6%) | 1.5 (4.5%) | 8 (1.2%) |
| Tianjin | 2.2 (3%) | 2.5 (0.6%) | 2.2 (2.1%) | 0.92 (6.3%) | 7.8 (1.3%) |
| Neimeng | 1.7 (2.7%) | 2.8 (1.5%) | 2.7 (3.7%) | 0.4 (3%) | 7.6 (2.3%) |
| Liaoning | 2.4 (1.9%) | 1.9 (0.43%) | 2 (1.7%) | 1.1 (3.7%) | 7.4 (1%) |
| Taiwan | 2.1 (0.37%) | 0.16 (0.01%) | 1.3 (0.18%) | 2.8 (1.2%) | 6.5 (0.22%) |
| Jilin | 2 (1.2%) | 1.3 (0.31%) | 1.4 (1.1%) | 0.69 (1.8%) | 5.3 (0.72%) |
| Fujian | 2.1 (0.36%) | 0.35 (0.05%) | 0.74 (0.25%) | 1.5 (0.69%) | 4.7 (0.26%) |
| Guangxi | 1.4 (0.23%) | 1.1 (0.15%) | 0.59 (0.18%) | 0.75 (0.38%) | 3.9 (0.2%) |
| Heilongjiang | 0.71 (0.53%) | 0.77 (0.21%) | 0.62 (0.49%) | 0.18 (0.56%) | 2.3 (0.35%) |
| Guangdong | 0.75 (0.12%) | 0.29 (0.04%) | 0.4 (0.13%) | 0.58 (0.31%) | 2 (0.11%) |
| Hongkong | 0.37 (nan%) | 0.17 (nan%) | 0.28 (nan%) | 0.43 (nan%) | 1.3 (nan%) |
| Hainan | 0.22 (0.06%) | 0.31 (0.04%) | 0.47 (0.08%) | 0.26 (0.27%) | 1.3 (0.07%) |
| TP | 40 (26%) | 129 (32%) | 46 (30%) | 7.1 (13%) | 221 (29%) |

Table S4. Precipitation contribution of TP ecosystems through T in different seasons (mm/season (%)). Numbers in parentheses denote relative contribution.

| **Region** | MAM | JJA | SON | DJF | Annual |
| --- | --- | --- | --- | --- | --- |
| Sichuan | 51 (19%) | 110 (18%) | 52 (16%) | 9.7 (13%) | 222 (17%) |
| Qinghai | 24 (25%) | 94 (33%) | 30 (26%) | 4.5 (18%) | 152 (29%) |
| Gansu | 24 (26%) | 37 (18%) | 22 (20%) | 4.1 (18%) | 87 (20%) |
| Xizang | 9.9 (5.6%) | 37 (7.8%) | 12 (7.7%) | 1.9 (3%) | 61 (7%) |
| Chongqing | 25 (6.4%) | 15 (2.9%) | 10 (2.9%) | 7.2 (6.2%) | 58 (4.2%) |
| Shaanxi | 19 (11%) | 10 (2.6%) | 12 (5.1%) | 4.8 (11%) | 46 (5.5%) |
| Ningxia | 11 (16%) | 13 (7.2%) | 14 (12%) | 2.6 (15%) | 41 (11%) |
| Guizhou | 11 (2.4%) | 7.3 (1.2%) | 3.9 (1.2%) | 3.5 (2.3%) | 26 (1.7%) |
| Hubei | 11 (3%) | 5.1 (0.88%) | 4 (1.4%) | 3.6 (3.2%) | 23 (1.8%) |
| Yunnan | 3.5 (1.2%) | 9.3 (1.1%) | 3.6 (1%) | 1 (0.92%) | 17 (1.1%) |
| Shanxi | 6.2 (6.3%) | 4 (1.2%) | 5.5 (3.5%) | 1.5 (6.5%) | 17 (2.9%) |
| Hunan | 6.4 (1.1%) | 2.3 (0.41%) | 1.5 (0.56%) | 2.1 (1.1%) | 12 (0.76%) |
| Henan | 4.6 (3.1%) | 2.1 (0.54%) | 2.9 (1.5%) | 1.9 (4.3%) | 11 (1.5%) |
| Anhui | 4.2 (1.3%) | 1.5 (0.24%) | 1.5 (0.64%) | 2.2 (1.7%) | 9.4 (0.72%) |
| Jiangxi | 4 (0.6%) | 0.96 (0.15%) | 0.94 (0.35%) | 1.6 (0.74%) | 7.5 (0.42%) |
| Zhejiang | 3.7 (0.77%) | 0.81 (0.11%) | 0.98 (0.31%) | 2 (0.92%) | 7.5 (0.43%) |
| Shanghai | 2.8 (0.87%) | 0.88 (0.15%) | 0.87 (0.35%) | 1.8 (1.1%) | 6.4 (0.48%) |
| Jiangsu | 2.4 (1.2%) | 1.1 (0.2%) | 1.1 (0.51%) | 1.6 (1.6%) | 6.3 (0.58%) |
| Hebei | 1.9 (2.3%) | 1.8 (0.51%) | 2 (1.7%) | 0.52 (3.5%) | 6.2 (1.1%) |
| Beijing | 1.5 (2.1%) | 1.8 (0.47%) | 1.9 (1.5%) | 0.45 (3.2%) | 5.6 (0.95%) |
| Shandong | 1.9 (1.7%) | 1.2 (0.3%) | 1.2 (1%) | 0.91 (2.7%) | 5.3 (0.77%) |
| Xinjiang | 1.1 (1.7%) | 2.8 (2.4%) | 0.87 (1.7%) | 0.18 (0.8%) | 4.9 (1.9%) |
| Tianjin | 1.5 (2%) | 1.5 (0.37%) | 1.3 (1.3%) | 0.5 (3.4%) | 4.8 (0.79%) |
| Taiwan | 1.6 (0.27%) | 0.12 (0.01%) | 0.91 (0.12%) | 1.9 (0.82%) | 4.5 (0.16%) |
| Neimeng | 1.1 (1.7%) | 1.6 (0.88%) | 1.6 (2.2%) | 0.18 (1.3%) | 4.5 (1.3%) |
| Liaoning | 1.5 (1.2%) | 1.2 (0.27%) | 1.2 (0.98%) | 0.53 (1.9%) | 4.4 (0.63%) |
| Fujian | 1.6 (0.27%) | 0.26 (0.04%) | 0.52 (0.18%) | 1 (0.49%) | 3.4 (0.19%) |
| Jilin | 1.2 (0.78%) | 0.78 (0.19%) | 0.79 (0.6%) | 0.32 (0.83%) | 3.1 (0.42%) |
| Guangxi | 1 (0.17%) | 0.83 (0.11%) | 0.42 (0.13%) | 0.55 (0.28%) | 2.8 (0.15%) |
| Guangdong | 0.55 (0.09%) | 0.21 (0.03%) | 0.28 (0.09%) | 0.42 (0.23%) | 1.5 (0.08%) |
| Heilongjiang | 0.45 (0.34%) | 0.45 (0.13%) | 0.34 (0.27%) | 0.08 (0.24%) | 1.3 (0.2%) |
| Hainan | 0.16 (0.04%) | 0.23 (0.03%) | 0.32 (0.06%) | 0.2 (0.2%) | 0.91 (0.05%) |
| Hongkong | 0.27 (nan%) | 0.13 (nan%) | 0.19 (nan%) | 0.31 (nan%) | 0.9 (nan%) |
| TP | 19 (12%) | 60.0 (15%) | 22 (14%) | 3.3 (6%) | 104 (14%) |

Table S5. Gross ecosystem changes in TP based on MODIS land cover data (MCD12Q1 V6) between 2001 to 2020. The change is expressed as the areal fraction of TP (%).

| Forest | Shrub | Grass | bare/snow | Other |
| --- | --- | --- | --- | --- |
| -0.29 | 0.80 | 0.10 | -0.60 | 0 |


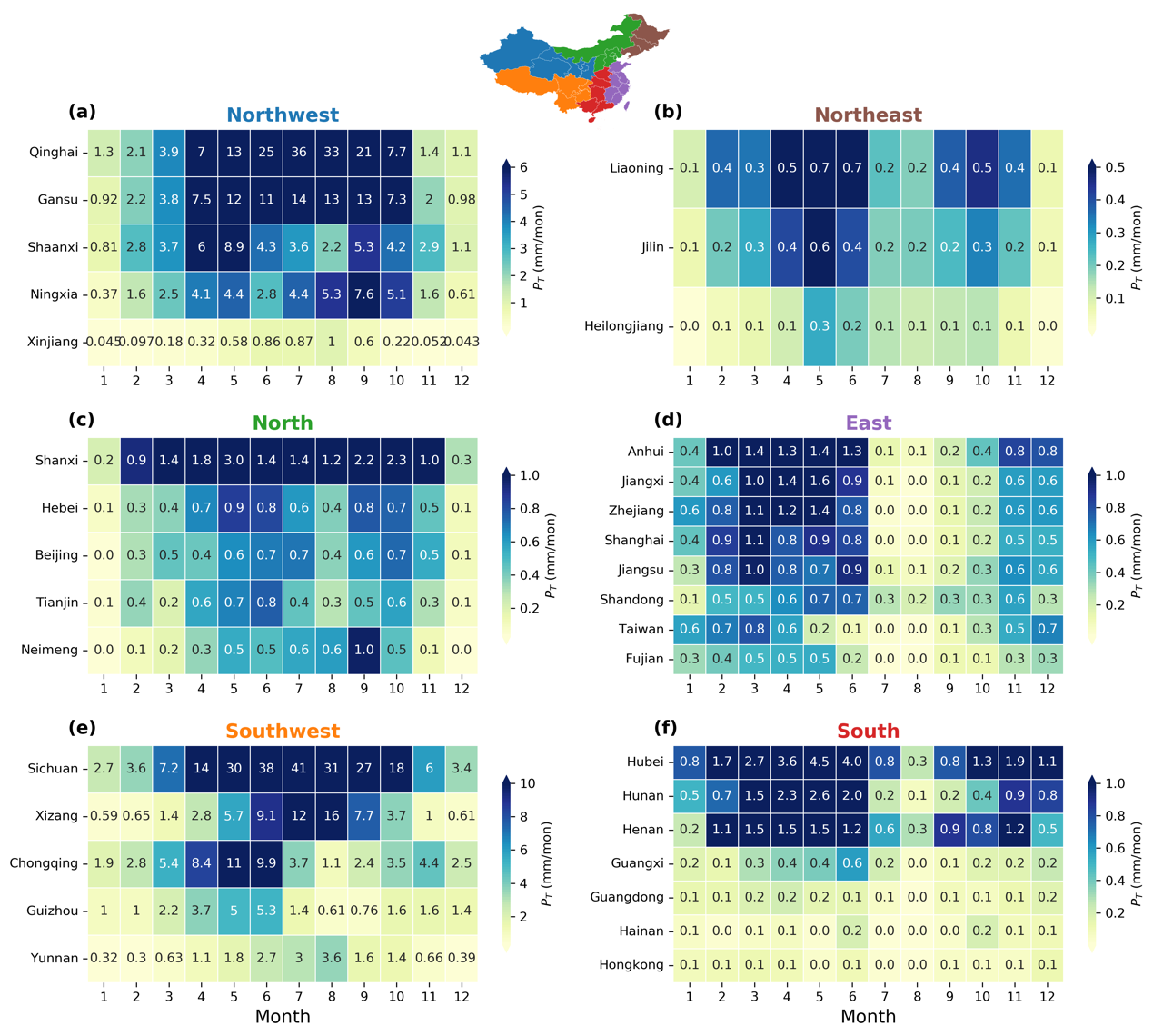


Figure S1. Seasonal variations in the precipitation contribution of TP ecosystems through T (P_T_) in China. Map lines delineate study areas and do not necessarily depict accepted national boundaries.


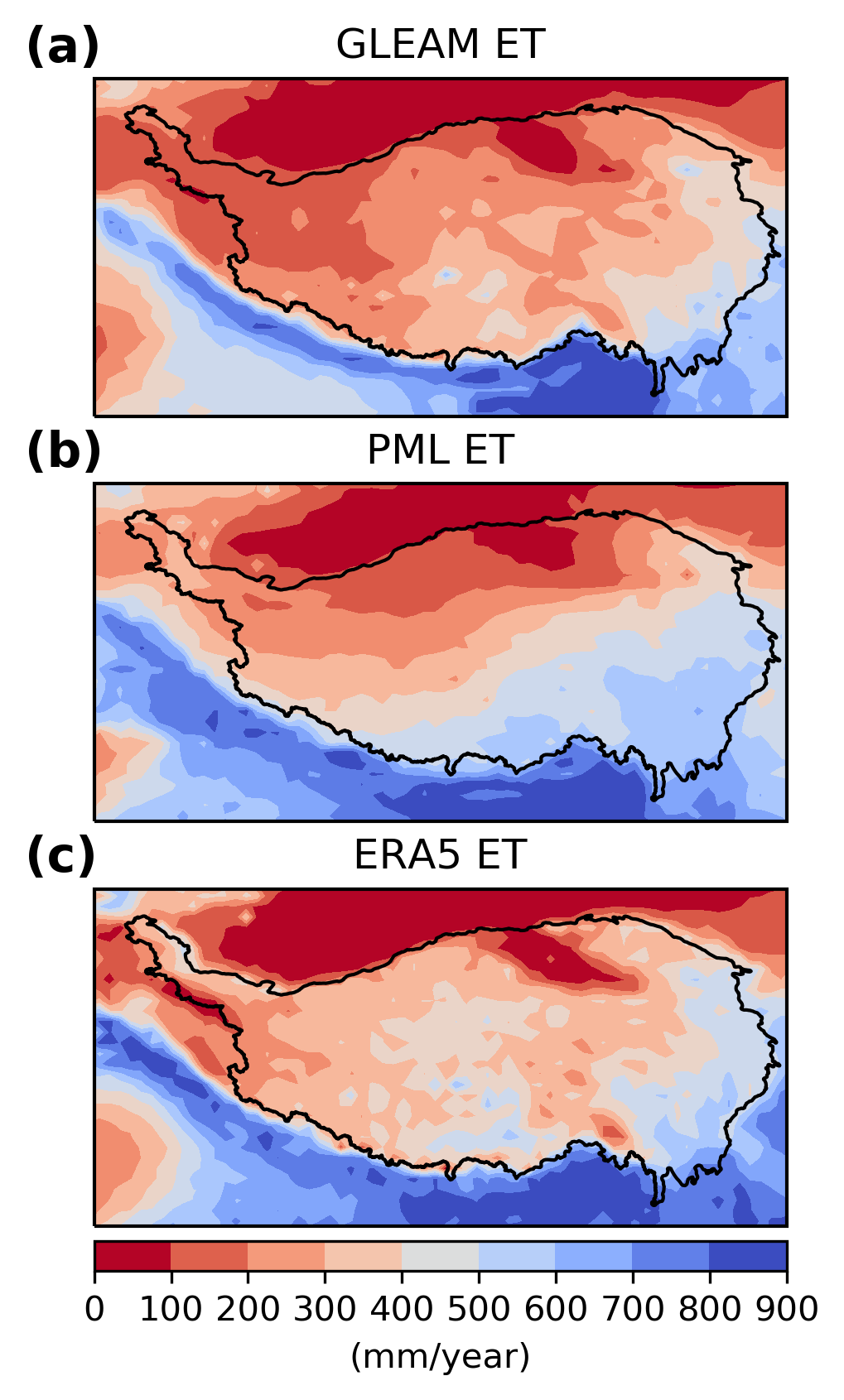


Figure S2. Comparison of ET data in TP from 2008 to 2017. Map lines delineate study areas and do not necessarily depict accepted national boundaries.
